# EIF4A3-mediated circ_0042881 activates the RAS pathway via miR-217/SOS1 axis to facilitate breast cancer progression

**DOI:** 10.1038/s41419-023-06085-4

**Published:** 2023-08-25

**Authors:** Chenxi Ju, Mingxia Zhou, Dan Du, Chang Wang, Jieqiong Yao, Hongle Li, Yang Luo, Fucheng He, Jing He

**Affiliations:** 1 Department of Medical Laboratory, The First Affiliated Hospital of Zhengzhou University, Zhengzhou, 450052 China; 2 Department of Gastroenterology, The First Affiliated Hospital of Zhengzhou University, Zhengzhou, 450052 China; 3 Department of Pathology, The Second Affiliated Hospital of Zhengzhou University, Zhengzhou, 450014 China; 4 Department of Molecular Pathology, The Affiliated Cancer Hospital of Zhengzhou University, Zhengzhou, 450008 China; 5 Center of Smart Laboratory and Molecular Medicine, School of Medicine, Chongqing University, Chongqing, 400044 China; 6 Department of Breast Surgery, The First Affiliated Hospital of Zhengzhou University, Zhengzhou, 450052 China

**Keywords:** Breast cancer, Tumour biomarkers

## Abstract

Breast cancer (BC) is one of the most frequent cancer-related deaths in women worldwide. Studies have shown the potential impact of circRNAs in multiple human tumorigeneses. Research on the vital signaling pathways and therapeutic targets of circRNAs is indispensable. Here, we aimed to investigate the clinical implications and underlying mechanisms of circ_0042881 in BC. RT-qPCR validated circ_0042881 was notably elevated in BC tissues and plasma, and closely associated with BC clinicopathological features. Functionally, circ_0042881 significantly accelerated the proliferation, migration, and invasion of BC cells in vitro and tumor growth and metastasis in vivo. Mechanistically, circ_0042881 promoted BC progression by sponging miR-217 to relieve its inhibition effect in son of sevenless 1 (SOS1), which further activated RAS protein and initiated downstream signaling cascades, including MEK/ERK pathway and PI3K/AKT pathway. We also demonstrated that treatment of BAY-293, an inhibitor of SOS1 and RAS interaction, attenuated BC progression induced by circ_0042881 overexpression. Furthermore, Eukaryotic initiation factor 4A-III (EIF4A3) could facilitate circ_0042881 circularization. Altogether, we proposed a novel signaling network in which circ_0042881, induced by EIF4A3, influences the process of BC tumorigenesis and metastasis by miR-217/SOS1 axis.

## Introduction

Breast cancer (BC), the most common type of cancer in women globally, has emerged as the main threat to women’s health. BC ranks first in the incidence rate and fifth in cancer mortality, accounting for 11.7% of all cancer cases worldwide and 6.9% of all cancer deaths, respectively [1]. It is estimated that there would be 287,850 new female BC cases and 51,400 deaths in 2022 in the United States [2]. The five-year survival rate for BC has considerably improved owing to the advances in early diagnosis, chemotherapy, endocrine therapy, and molecular targeted therapy in the last few decades. However, BC continues to lead the pack in the vast majority of nations for mortality [3]. Additionally, the frequency of BC recurrence keeps increasing [2]. Therefore, it is urgent to explore more specific and effective diagnostic biomarkers and therapeutic targets for the treatment of BC.

Circular RNAs (circRNAs) have recently been mentioned in numerous research studies owing to their distinct impact on the development of cancers, including BC. Compared with linear mRNAs, circRNAs possess peculiar covalently closed loop structures without 5’ caps and 3’ tails, which hold responsibility for those intracellular stability [4]. CircRNAs are known to be evolutionarily conserved in different species [5] and exhibit cell-specific or tissue-specific [6]. With these factors in mind, circRNAs may play roles as a novel type of tumor biomarkers. The molecular function of circRNAs has been identified in both the transcriptional and post-transcriptional regulation of cell proliferation, apoptosis and tumor metastasis through serving as microRNA (miRNA) sponge [7], regulating parental transcription and translation [8], interacting with proteins [9] and existing as protein translation templates [10]. Their expression patterns in various tumor tissues reflect tumor size, lymphatic metastasis, TNM stage, and prognosis. Based on the above functions, emerging circRNAs have been demonstrated to play a pivotal role in multiple human diseases, especially cancers.

Circ_0042881, also known as circNF1, is generated by head-to-tail splicing of exons 2 to 8 of the neurofibromin 1 (NF1) transcript. According to the previous reports, circ_0042881 functioned as a sponge for miR-340 and enhanced the proliferation of glioblastoma cells [11]. In gastric carcinoma, circ_0042881 induced the malignant development of tumor cells through serving as miR-16 sponge to depress its target genes, the microtubule-associated protein 7 (MAP7) and AKT serine/threonine kinase 3 (AKT3) [12]. The expression and function of circ_0042881, however, have not been illustrated, and their specific mechanism has yet to be characterized in BC.

Most commonly understood as post-transcriptional regulators, miRNAs attach to the 3’ untranslated region (UTR) of their target mRNAs to govern mRNA degradation or translation suppression [13]. According to growing data, the majority of circRNAs have a role in the development of BC by acting as miRNA sponges. CircRNF10, for instance, may prevent the progression of BC by sponging miR-934 and controlling the PTEN/PI3K/AKT axis [14]. Similarly, circ-TRIO could sponge miR-432-5p to regulate the expression of Coiled-Coil Domain Containing 58 (CCDC58) and participate in triple negative BC tumorigenesis [15].

To discover novel diagnostic biomarkers and elucidate the molecular mechanism of BC progression, we uncovered a recently identified circRNA, termed as circ_0042881, which has not been previously investigated in BC. Circ_0042881 was elevated in BC tissues and cell lines, and boosted the proliferation, migration, and invasion of BC cells, as evidenced by in vitro and in vivo functional experiments. Furthermore, we illustrated the underlying mechanism of son of sevenless 1 (SOS1) regulation at the post-transcriptional stage in BC by circ_0042881 functioning as a competitive endogenous RNA (ceRNA). Importantly, NF1 pre-mRNA contained Eukaryotic initiation factor 4A-III (EIF4A3) binding sites, which could promote the biogenesis of circ_0042881. Taken together, our study broadens the knowledge of the pathophysiology of BC and may serve as a prognostic biomarker or as a potential therapeutic target for BC patients.

## Result

### The expression and characterization of circ_0042881 in breast cancer

**Fig. 1:**
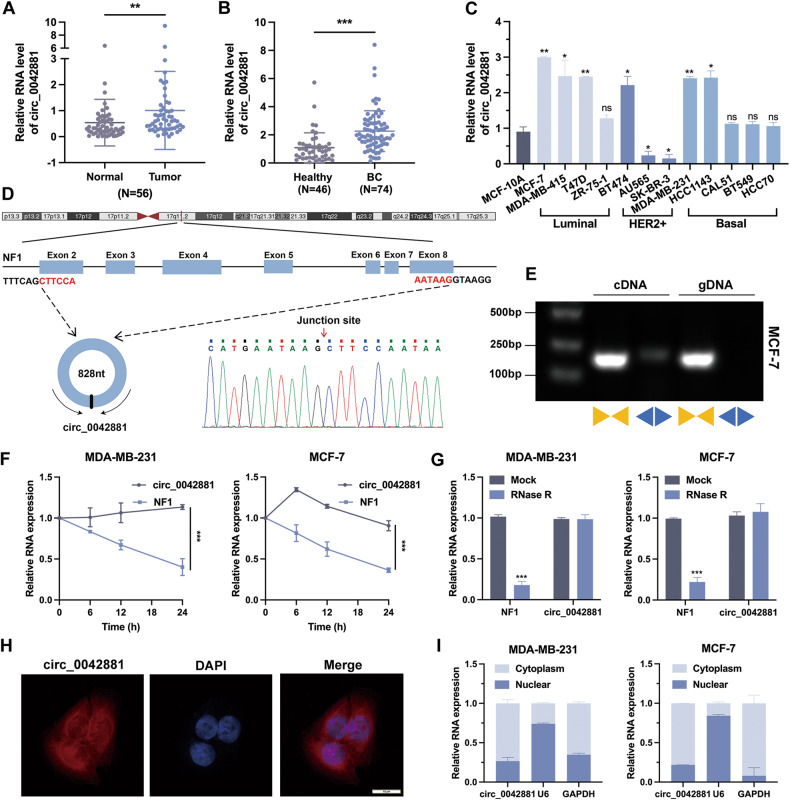
Circ_0042881 is highly expressed in breast cancer. **A** RT-qPCR analysis of the relative circ_0042881 expression in 56 pairs of BC tissues (Tumor) and adjacent non-tumor tissues (Normal). **B** RT-qPCR analysis of the relative circ_0042881 expression in plasma of BC patients (*N* = 74) and healthy donors (*N* = 46). **C** Relative circ_0042881 expression in BC cell lines and normal mammary epithelial cell line (MCF-10A) was determined by RT-qPCR. **D** Schematic illustration of circ_0042881 location and formation, with the junction site clarified by Sanger sequencing. **E** PCR with convergent and divergent primers and agarose gel electrophoresis to verify the circular structure of circ_0042881 in MCF-7; divergent and convergent primers are indicated by the direction of the arrow. **F**, **G** The relative expression of NF1 and circ_0042881 after treated with Actinomycin D and RNase R were determined by RT-qPCR. **H** Representative FISH images of circ_0042881 were shown. The circ_0042881 probe was labeled with Cy3 (red). The nuclei were stained with DAPI (blue). (Magnification x 1000. Scale bar = 10 μm). **I** Nuclear-cytoplasmic fractionation assay showed the percentage of circ_0042881 levels in nucleus and cytoplasm. U6 was used as the nucleus reference and GAPDH was used as the cytoplasmic reference. The data are presented as mean ± SD. *P < 0.05, **P < 0.01, ***P < 0.001.

In order to determine the potential role of newly identified circ_0042881 in BC, RT-qPCR assay was adopted on 56 pairs of BC tissues and adjacent non-tumor tissues, which demonstrated that the expression level of circ_0042881 was robustly higher in BC tissues (Fig. 1A). Patients were grouped according to the median expression value of circ_0042881. Association between clinicopathological features and circ_0042881 expression was analyzed and shown in Table S1. Patients with high circ_0042881 profiles possessed more advanced TNM stages and greater tumor sizes. Due to the distinctive circular structure, circRNAs were discovered to be persistently expressed in plasma, and their long half‐life makes them appropriate biomarkers. To evaluate the diagnostic value of circ_0042881, we examined their level in plasma. The results revealed a substantial elevation of circ_0042881 in BC patients’ plasma than healthy controls (Fig. 1B). Following the construction of the receiver operating characteristic (ROC) curve, the area under the ROC curve (AUC) was calculated to be 0.802, indicating that circ 0042881 may have prospect as non-invasive biomarkers for detection of BC (Fig. S1A). Moreover, the overexpression of circ_0042881 was further validated in BC cell lines (Fig. 1C). Compared with normal mammary epithelial cell line MCF-10A, circ_0042881 presented relatively elevated level in multiple BC cell lines, particularly in MCF-7 and MDA-MB-231 cells. We decided to employ those two cell lines in subsequent research.

According to the circBase database, circ_0042881 is located on chromosome 17, and generated by head-to-tail splicing of exons 2 to 8 of the NF1 transcript. We confirmed the sequence of the back-splicing junction point using Sanger sequencing, and it coincided with the circBase annotation (Fig. 1D). In addition, the convergent and divergent primers were designed and synthesized to perform PCR and agarose gel electrophoresis. As shown in Figs. 1E and S1B, the convergent primers could incorporate with NF1 linear mRNA and genomic DNA to initiate PCR process, while the divergent primers could only amplify in the existence of circ_0042881. Actinomycin D can interfere the process of cellular transcription to prevent mRNA synthesis. We used it to verify the stability of circ_0042881. The results demonstrated that the half-life of circ_0042881 was longer than 24 h, which was more stable than its parental mRNA (Fig. 1F). Besides, due to the distinct cyclic structure, the expression of circ_0042881 cannot be affected by RNase R treatment (Fig. 1G). We performed nuclear-cytoplasmic separation assay and fluorescence in situ hybridization (FISH) to determine the subcellular localization of circ_0042881, confirming that it was predominantly distributed in the cytoplasm of BC cells (Fig. 1H, I). According to the aforementioned findings, circ_0042881 is a circular RNA that is mainly found in the cytoplasm of BC cells. Its high expression level in tumor tissues and plasma suggests that it may be a viable biomarker for BC.

### Circ_0042881 promotes BC cell proliferation, migration and invasion in vitro

**Fig. 2:**
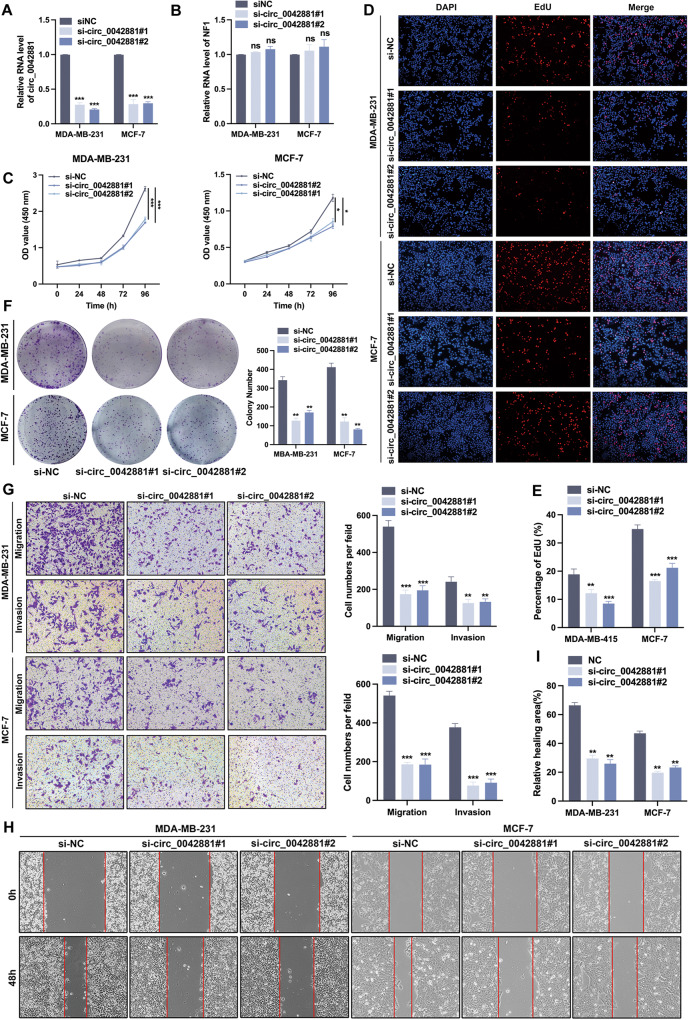
Knockdown of circ_0042881 suppresses BC progression. **A**, **B** RT-qPCR analysis of the relative circ_0042881 and NF1 expression after circ_0042881 knockdown in MDA-MB-231 and MCF-7. **C−F** The effect of silence circ_0042881 on proliferation in MDA-MB-231 and MCF-7 was tested by CCK-8, EdU and colony formation assays. **G** Transwell assays were performed to analyze cell migration and invasion ability. **H**, **I** Wound healing assays were used to evaluate the cell migration ability of each group. The data are presented as mean ± SD. *P < 0.05, **P < 0.01, ***P < 0.001.

To investigate the function of circ_0042881 in BC cells, we used two siRNAs (si-circ_0042881#1, si-circ_0042881#2) to deplete circ_0042881 in BC cells. We also constructed the lentivirus vector to establish persistently overexpressed cell lines of circ_0042881. Transfection efficiency was verified by RT-qPCR (Fig. 2A, B, Fig. S2A). The expression of circ_0042881 was downregulated and overexpressed by indicated plasmids in BC cells, while the expression of NF1 did not alter, suggesting that circ_0042881 knockdown did not equally affect its parental mRNA transcription. Then cell counting kit-8 (CCK-8) assay revealed that knockdown of circ_0042881 significantly restricted cell growth, while cell viability was accelerated after circ_0042881 overexpression (Fig. 2C, S2B). Besides, the 5-ethynyl-2’-deoxyuridine (EdU) assay and colony formation experiments proved that the proliferation abilities of MDA-MB-231 and MCF-7 were reduced after circ_0042881 knocking-down, while overexpression of circ_0042881 provoked the phenotypes (Fig. 2D−F, Fig. S2C−E). We further assessed the alteration of migration and invasion abilities by transwell assay, the number of migrant and invasive cells exhibited an obvious reduction in si-circ_0042881 group, and was increased in the circ_0042881 overexpressed group (Fig. 2G, Fig. S2F). In wound healing assay, we observed a distinct change in relative healing areas after circ_0042881 knocked down and overexpressed in MDA-MB-231 and MCF-7 cells (Fig. 2H, I, Fig. S2G). Taken together, those data suggested that circ_0042881 could aggravate the proliferation, migration, and invasion of BC cells in vitro.

### Circ_0042881 functions as a sponge of miR-217

**Fig. 3:**
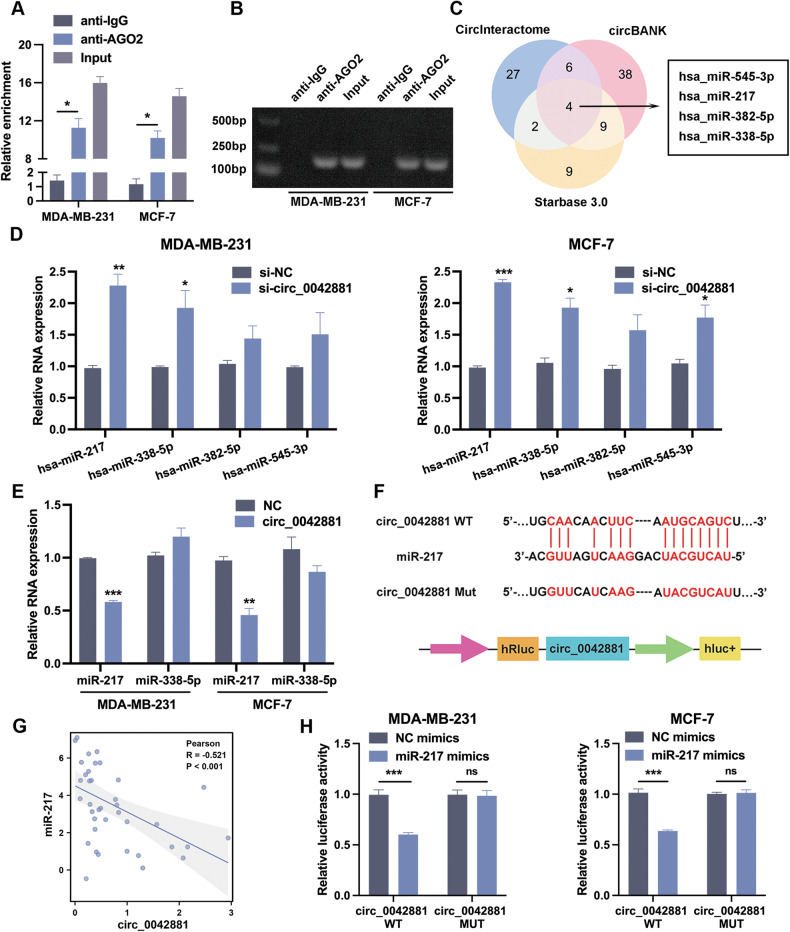
Circ_0042881 serves as a miR-217 sponge. **A**, **B** RIP experiments were performed, and RT‐qPCR assays and agarose gel electrophoresis were used to detect the enrichment of circ_0042881 to AGO2. **C** Venn diagram representing the potential targeted miRNAs of circ_0042881 by CircInteractome, circBANK, Starbase v3.0. **D** RT-qPCR analyzed the relative expression of 4 candidate miRNAs in MDA-MB-231 and MCF-7 after transfecting with si-circ_0042881. **E** The relative expression of miR-217 and miR-338-5p was tested by RT-qPCR after circ_0042881 overexpression. **F** To examine the direct interaction between circ_0042881 and miR-217, the luciferase reporter vectors containing circ_0042881 WT or circ_0042881 MUT were constructed. **G** The correlation between circ_0042881 and miR-217 in BC tissues was analyzed by Pearson correlation analysis. **H** The luciferase activities in MDA-MB-231 and MCF-7 cells co-transfected with miR-217 mimic or mimic NC and luciferase reporters containing circ_0042881 WT or circ_0042881 MUT. The data are presented as mean ± SD. *P < 0.05, **P < 0.01, ***P < 0.001.

Given that circ_0042881 is an exonic circRNA and predominantly located in the cellular cytoplasm, which led us to speculate whether it would act as a miRNA sponge to control gene expression [16]. In order to ascertain the miRNA binding accessibility of circ_0042881, we first carried out the RNA immunoprecipitation (RIP) assay using anti-AGO2. As illustrated in Fig. 3A, B, circ_0042881 was obviously enriched by AGO2 antibody compared to anti-IgG. Using online bioinformatics databases (CircInteractome, circBANK, Starbase v3.0), we next predicted the potential target miRNAs of circ_0042881, and four candidate miRNAs (hsa_miR-545-3p, hsa_miR-338-5p, hsa_miR-382-5p, hsa_miR-217) were screened out (Fig. 3C). RT-qPCR was subjected to further confirmation. Strikingly, miR-217 and miR-338-5p were simultaneously upregulated in MDA-231 and MCF-7 cells following circ_0042881 knockdown (Fig. 3D). Meanwhile, only miR-217 was declined in circ_0042881 overexpression group (Fig. 3E). Thus, we assumed that circ_0042881 may serve as a miR-217 sponge in BC. To validate this hypothesis, we constructed circ_0042881 wild-type and the miR-217-binding-site mutated luciferase reporter to testify the regulation relationship of circ_0042881 and miR-217 (Fig. 3F). As shown in Fig. 3H, the luciferase activity was significantly suppressed when cells were co-transfected with miR-217 mimic and the circ_0042881 wild-type luciferase reporter, whereas the luciferase activity showed no obvious difference when cells were co-transfected miR-217 mimic with the circ_0042881 mutant luciferase reporter. The correlation analysis was performed by Pearson’s correlation test, which confirmed a negative correlation between circ_0042881 and miR-217 expression in 37 pairs of BC tumor tissues (Fig. 3G). Overall, those observations implied that circ_0042881 and miR-217 can interact directly.

### Circ_0042881 aggravates BC progression by sponging miR-217

**Fig. 4:**
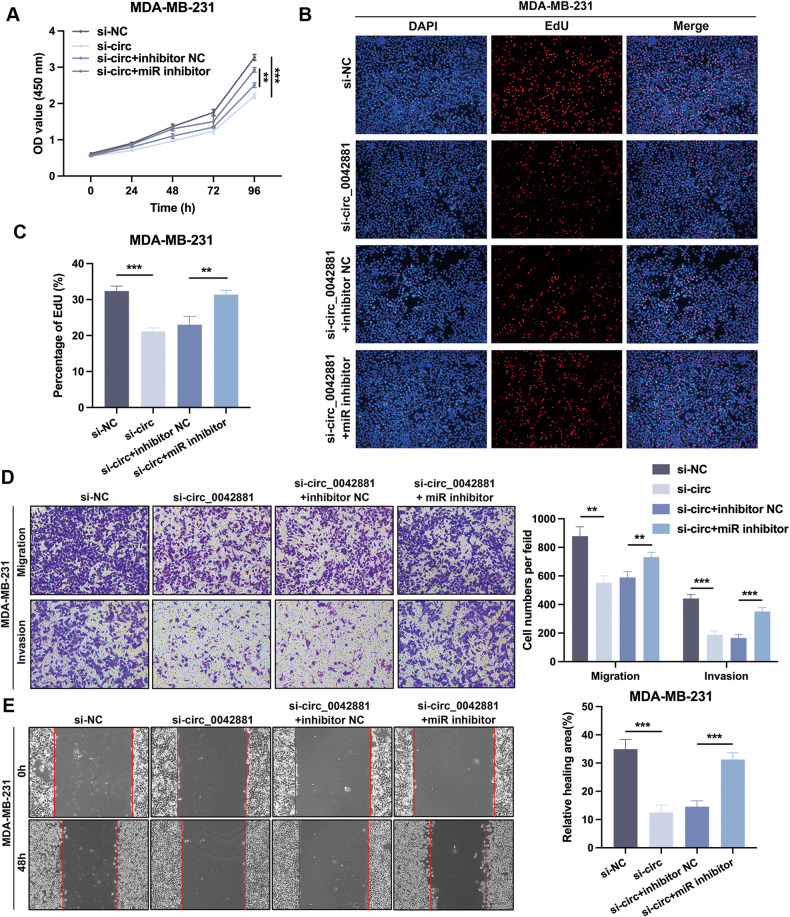
The tumor-inhibition effects of si-circ_0042881 could be reversed by miR-217 inhibitor. **A**−**C** CCK-8, EdU assays were performed to assess cell proliferation ability of each group. **D** The capacity of cell migration and invasion of each group was determined by transwell assays. **E** The effect of si-circ_0042881 and miR-217 inhibitor on migration was examined by wound healing assays. The data are presented as mean ± SD. **P < 0.01, ***P < 0.001.

The rescue experiments were conducted to further explore the influence of circ_0042881 and miR-217 on BC progression. MDA-MB-231 and MCF-7 cells were co-transfected with si-circ_0042881 and miR-217 inhibitors. As depicted in Fig. 4A, the decreasing effects of circ_0042881 inhibition on MDA-MB-231 growth could be abolished by miR-217 inhibitors. Similar results were disclosed in MCF-7 cells (Fig. S3A). Furthermore, EdU assay revealed that miR-217 inhibitors reversed the suppression effect of si-circ_0042881 on BC cells EdU‐positive cell rate (Fig. 4B, C, Fig. S3B, C). Moreover, circ_0042881 depletion markedly repressed the migration and invasion of BC cells in transwell and wound healing assays, whereas the inhibitory effects could be overturned by miR-217 inhibitors (Fig. 4D, E, Fig. S3D, E). Above all, those results pointed out that circ_0042881 serves as a sponge for miR-217 to promote BC cell malignant behaviors.

### SOS1 is a direct target of miR-217

**Fig. 5:**
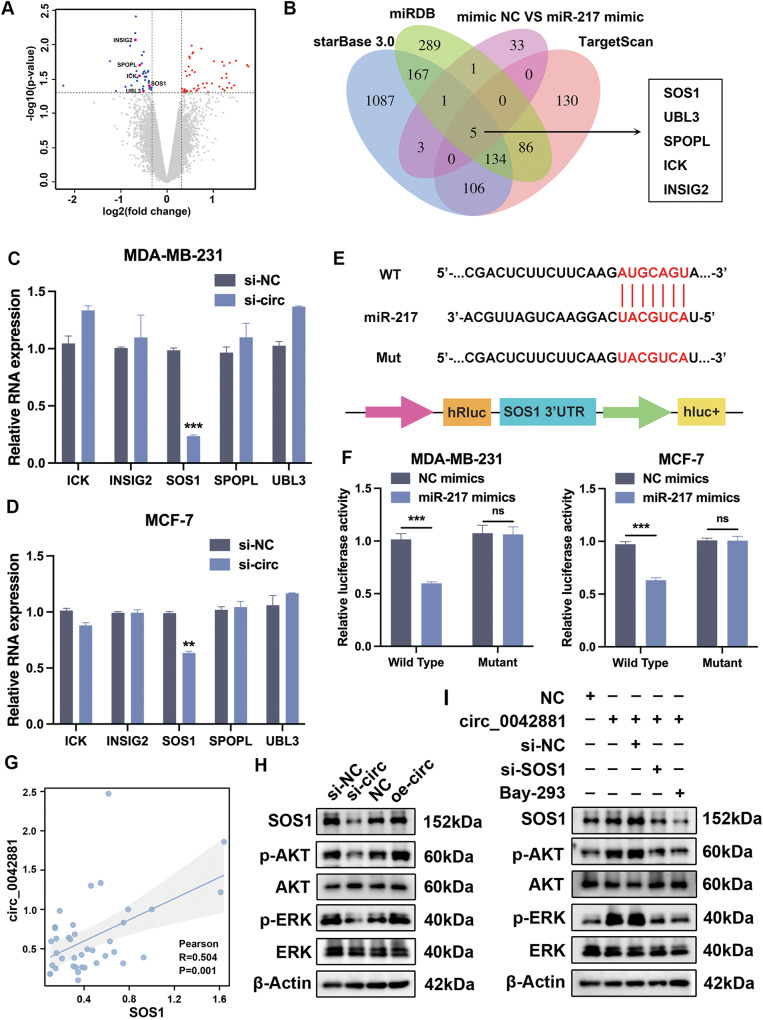
SOS1 is targeted by miR-217. **A** Volcano plot showing differentially expressed genes in MDA-MB-231 cells transfected with mimic NC and miR-217 mimic. **B** Venn diagram representing the potential targeted mRNAs of miR-217 by miRDB, TargetScan, Starbase v3.0 and RNA-seq results. **C**, **D** RT-qPCR analyzed the relative expression of 5 candidate mRNAs in MDA-MB-231 and MCF-7 after transfecting with si-circ_0042881. **E**, **F** The luciferase activities in MDA-MB-231 and MCF-7 cells co-transfected with miR-217 mimic or mimic NC and luciferase reporters containing SOS1 3’UTR WT or SOS1 3’UTR MUT. **G** The correlation between circ_0042881 and SOS1 in BC tissues was analyzed by Pearson correlation analysis. **H**, **I** Western blotting analyed the protein levels of SOS1, AKT, p-AKT, ERK, and p-ERK after corresponding treatment. The data are presented as mean ± SD. **P < 0.01, ***P < 0.001.

To ascertain the potential downstream target genes of miR-217, RNA-seq was utilized to explore the differentially expressed transcriptomes between MDA-MB-231 transfected with mimic NC and miR-217 mimic, which identified 45 upregulated genes and 43 downregulated genes (Fig. 5A). The downregulated genes were overlapped with the predicted target genes of miR-217 from the Starbase 3.0, miRDB and TargetScan databases (Fig. 5B). A total of 5 genes were then selected and RT-qPCR analysis was conducted for further evaluation. Intriguingly, only SOS1 was profoundly reduced by circ_0042881 knockdown, and overexpression of circ_0042881 elevated its expression in MDA-MB-231 and MCF-7 cells (Fig. 5C, D, Fig. S4C). In order to find out whether miR-217 could directly bind to the 3’UTR of SOS1, a dual-luciferase reporter experiment was conducated. The miR-217 mimic strikingly inhibited the luciferase activity of cells containing wild type SOS1 3’UTR, while no significant change was observed in SOS1 binding site mutant cells (Fig. 5E, F). As expected, among patients with high expression of circ_0042881, the expression of SOS1 appeared to increase in BC tissues (Fig. S4D). Subsequent RT-qPCR reflected the positive correlation between SOS1 and circ_0042881 expression levels in BC specimens (Fig. 5G).

SOS1 is a guanine nucleotide exchange protein, and exerts indispensable role in the activation of RAS protein [17], which then stimulates the downstream pathways including the MEK/ERK pathway and PI3K/AKT pathway to facilitate tumor cell survival [18]. Moreover, it was discovered by Kyoto encyclopedia of genes and genomes (KEGG) and gene set enrichment analysis (GSEA) enrichment analysis that the target genes for miR-217 are associated to the RAS signaling pathway (Fig. S4B, E). Hence, we set out to explore whether circ_0042881 could be effective in the activation of MEK/ERK pathway and PI3K/AKT pathway. To this end, western blotting was operated after circ_0042881 knockdown and overexpression, and we discovered that the level of phosphorylated AKT and ERK was suppressed in MDA-MB-231 cells with low circ_0042881 expression (Fig. 5H). The increased SOS1 following circ_0042881 overexpression was accompanied by an increase in AKT and ERK phosphorylation, however, SOS1 gene silencing nullified this effect (Fig. 5I). To further investigate whether AKT and ERK phosphorylation are induced by SOS1/RAS pathway, we used BAY-293 (SOS1/RAS interaction inhibitor) to treat cells. As anticipated, BAY-293 significantly reduced the levels of AKT and ERK phosphorylation that were brought on by circ_0042881 (Fig. 5I). Consistently, we verified that circ_0042881 could elevate the expression of SOS1 by sponging miR-217, which then induced RAS protein to promote the phosphorylation of AKT and ERK, ultimately causing the activation of MEK/ERK and PI3K/AKT pathway.

### Circ_0042881 expedites BC cell proliferation and metastasis through the SOS1/RAS pathway

**Fig. 6:**
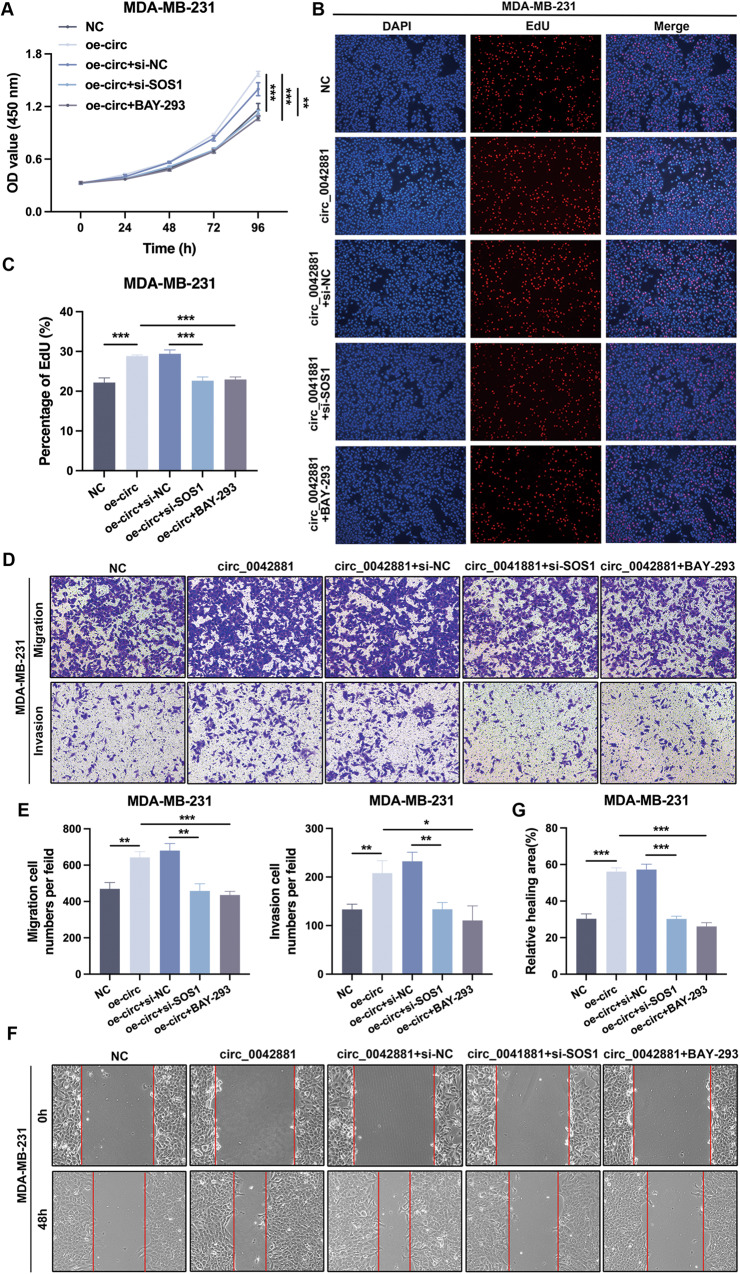
Circ_0042881 promotes proliferation and metastasis in a SOS1‐dependent manner. **A**−**C** CCK-8, EdU assays were performed to assess cell proliferation ability of each group. **D**, **E** The capacity of cell migration and invasion of each group was determined by transwell assays. **F**, **G** The effect of circ_0042881 overexpression, si-circ_0042881 and BAY-293 treatment on migration was examined by wound healing assays. The data are presented as mean ± SD. **P < 0.01, ***P < 0.001.

To investigate whether circ_0042881 positively regulated the malignant behaviors of BC cells through SOS1/RAS pathway, a series of rescue experiments were employed. CCK-8 assay confirmed that ectopic expression of circ_0042881 assisted the proliferation of BC cells. The facilitative effect was significantly reversed when we simultaneously inhibited SOS1 expression or treated cells with BAY-293 (Fig. 6A, Fig. S5A). Notably, the EdU assay produced comparable results. The proliferation ability of BC cells boosted by circ_0042881 upregulation was abrogated by SOS1 silencing or blocking the interaction of SOS1 and RAS using BAY-293 (Fig. 6B, C, Fig. S5B, C). Furthermore, results from transwell assay and wound healing assay corroborated the conclusion that upregulating circ_0042881 was favorable for BC cells migration and invasion, while these capacities of cells in the presence of si-SOS1 or BAY-293 were not influenced sensibly (Fig. 6D−G, Fig. S5D−G). Next, we asked whether SOS1 overexpression could reverse the suppressive effect of circ_0042881 knockdown on malignant phenotypes. The rescue assays demonstrated that the restrictive effects of circ_0042881 knockdown on cell proliferation, migration and invasion can be readily reversed by ectopic expression of SOS1 (Fig. S7−S8). In conclusion, we confirmed that circ_0042881 could boost BC cell proliferation, invasion, and migration by activating SOS1/RAS signaling.

### EIF4A3 functions as an upstream target of circ_0042881

**Fig. 7:**
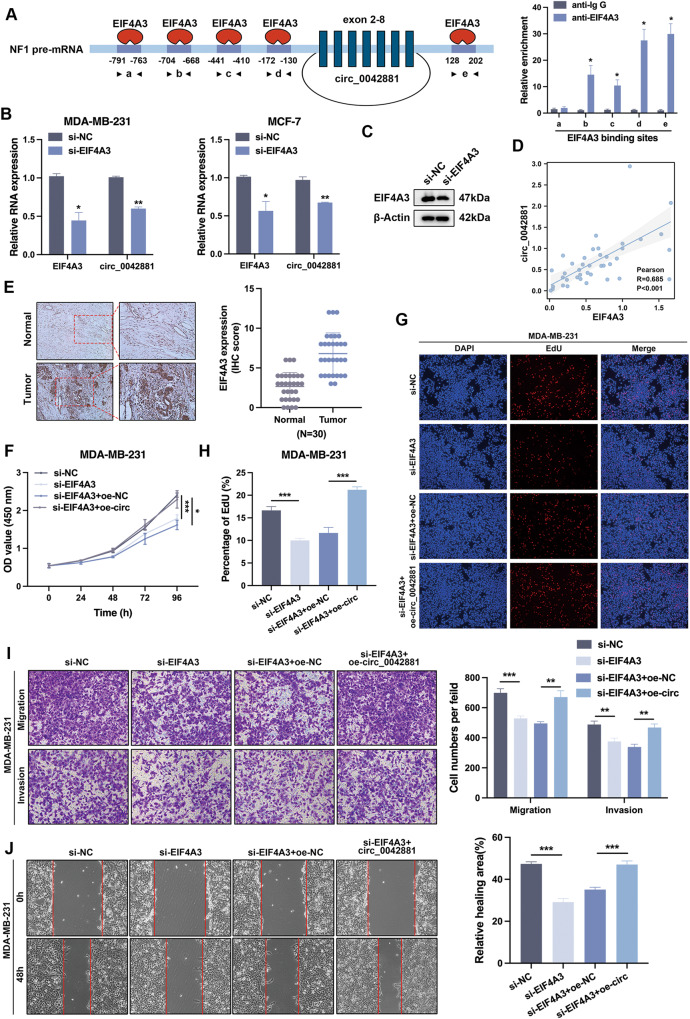
RNA binding protein EIF4A3 regulates the expression of circ_0042881. **A** The binding sites for EIF4A3 in the flanking sequences of the NF1 pre-mRNA transcript were predicted using CircInteractome and RIP assays validated the binding sites between NF1 and circ_0042881. **B** The relative expression of EIF4A3 and circ_0042881 after EIF4A3 knockdown was detected by RT-qPCR. **C** The protein level of EIF4A3 after EIF4A3 knockdown was detected by western blotting. **D** The correlation between circ_0042881 and EIF4A3 in BC tissues was analyzed by Pearson correlation analysis. **E** Representative images of immunohistostaining for EIF4A3 of tumor and adjacent non-tumor tissues (*n* = 30). **F**−**H** The proliferation of MDA-MB-231 was detected by CCK-8 and EdU assays. **I**, **J** Representative results of transwell and wound healing assays of each group.

According to previous studies, a number of RNA binding proteins (RBPs), including Quaking (QKI), Fused in Sarcoma (FUS) and EIF4A3, can either accelerate or suppress the back-splicing activity to impact the synthesis of circRNAs [19–21]. To further analyze whether circ_0042881 could be regulated by RBPs, the putative RBPs binding sites were predicted in circ_0042881. We found that EIF4A3 harbored the most number of binding sites, as indicated by CircInteractome. RIP assay indicated that EIF4A3 could combine with flanking sequences of NF1 pre-mRNA in 4 putative binding sites (Fig. 7A). We knocked down EIF4A3 in MDA-MB-231 and MCF-7 in order to determine the expression change of circ_0042881 in response to EIF4A3 downregulation. The knockdown efficiency was then confirmed by western blotting and RT-qPCR (Fig. 7B, C). As shown in Fig. 7B, circ_0042881 expression could be inhibited by a deficiency of EIF4A3. Furthermore, RT-qPCR of 39 paired BC tissues revealed that circ_0042881 and relative EIF4A3 expression were positively associated (Fig. 7D). And immunohistochemistry analysis displayed higher expression level of EIF4A3 in BC tissues compared with adjacent non-tumor tissues (Fig. 7E). Analysis from Kaplan-Meier plot offered that the higher expression level of EIF4A3 was correlated with shorter overall survival (OS) (Fig. S8A).

To further examine the interplay between EIF4A3 and circ_0042881, we performed rescue experiments to evaluate the biological effects of BC cells co-transfected with si-EIF4A3 and/or circ_0042881 overexpression. According to the CCK-8, EdU assays results, blocking EIF4A3 vividly repressed cell growth. However, simultaneous overexpression of circ_0042881 partially reverse the negative effects (Fig. 7F−H, Fig. S8B−D). In addition, the migration and invasion abilities of MDA-MB-231 and MCF-7 were tested by transwell and wound healing assays. As depicted in Fig. 7I, J and Fig. S8E−H, inhibition of EIF4A3 impaired those malignant phenotypes, whereas overexpression of circ_0042881 elicited a total changeover effect. In summary, EIF4A3 could promote the back-splicing process of circ_0042881 and expedite BC malignant progression.

### Circ_0042881 accelerates tumorigenesis and metastasis of BC cells in vivo

**Fig. 8:**
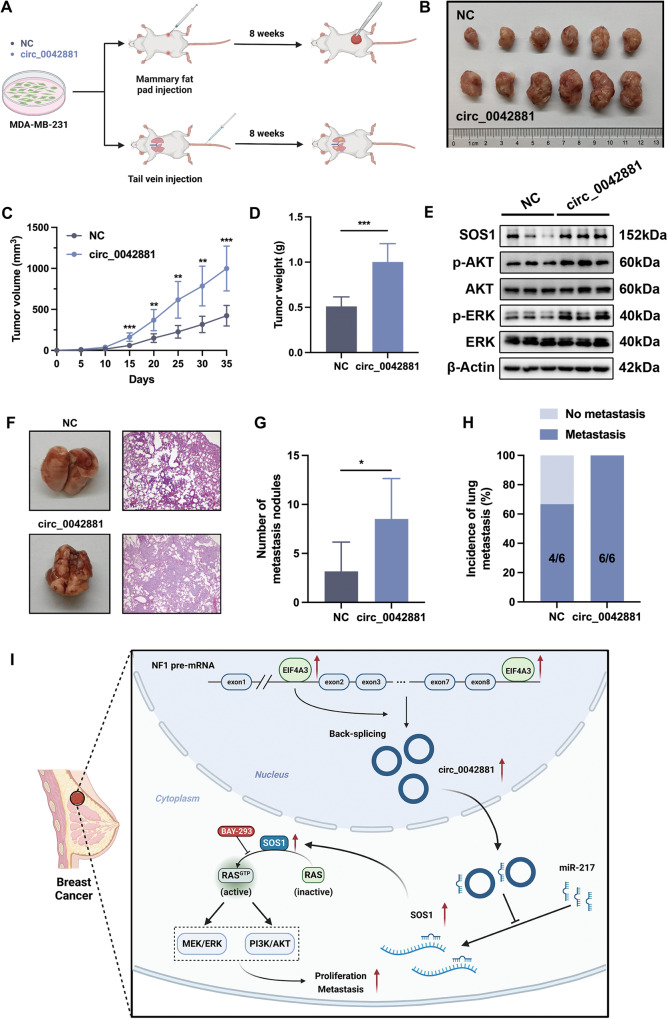
Circ_0042881 promotes the growth and metastasis of BC in vivo. **A** Schema of the BC orthotopic mouse models and metastatic models. **B** Representative images of BC orthotopic mouse models in NC and circ_0042881 groups. **C** Tumor volume was measured every 5 days. **D** Tumor weight was examined in each group. **E** The protein levels of SOS1, AKT, p-AKT, ERK, and p-ERK in each group were verified by western blotting. **F** Representative images of lung tissues and their corresponding hematoxylin and eosin-stained sections in BC metastatic models. **G**, **H** The number of metastasis nodules and the incidence of lung metastasis were measured in NC and circ_0042881 groups. **I** Schematic depiction of the mechanism underlying EIF4A3-mediated circ_0042881 promotes BC progression by miR-217/SOS1 axis.

To evaluate the role of circ_0042881 in BC progression, we firstly performed tumorigenesis assays using orthotopic BC mouse model. Twelve NOD/SCID mice were divided into two groups, either control or circ_0042881 overexpressing MDA-MB-231 cells were implanted into the fourth mammary fat pad of NOD/SCID mice (Fig. 8A). Of note, ectopic expression of circ_0042881 resulted in the faster tumor formation of the orthotopic xenografts, which was consistent with the in vitro findings (Fig. 8B−D). The western blotting results showed that the expression of p-AKT and p-ERK was increased in tumor tissues with ectopic circ_0042881 expression (Fig. 8E). We then asked whether circ_0042881 is essential for the metastasis-promoting effects of BC cells in vivo, experimental lung metastasis models were established (Fig. 8A). Intriguingly, MDA-MB-231 cells with circ_0042881 overexpression generated larger numbers of metastatic lung nodules (Fig. 8F, G). Importantly, the incidence of pulmonary metastasis in the circ_0042881 overexpression group was 100%, while the incidence of lung metastasis in control group was only 66.7% (4/6) (Fig. 8H). In summary, these in vivo findings complement the in vitro results, underscoring the critical roles of circ_0042881 in BC growth and metastasis (Fig. 8I).

## Discussion

As one kind of novel non-coding RNAs, circRNAs are widely recognized as essential components of cancer-related biological processes formed by back-splicing [22, 23]. Additionally, the features of aberrant expression in diversified cancers and structural stability provide circRNAs critical advantages to emerge as the targets for cancer diagnosis and treatment. In BC, there is a substantial body of evidence that circRNAs mediate the occurrence, development and malignant progression [24]. Ling et al. discovered that up-regulation of circCDYL2 could mediate the inhibition of growth factor receptor-bound protein 7 (GRB7) ubiquitination to improve the formation of GRB7- focal adhesion kinase (FAK) complex, thereby inducing tumorigenesis and trastuzumab resistance via aberrantly activating downstream AKT and ERK1/2 signaling pathway [25]. CircSEMA4B exerted its tumor suppressive effects by encoding a novel protein SEMA4B-211aa, which could hinder the phosphorylation of AKT through regulating the formation of second messenger PIP3 [10]. Additionally, it was reported that circNOLC1 contributed the improvement of cancer stem cell functions in BC through miR-365a-3p/STAT3 axis [26]. Herein, for the first time, we found that circ_0042881 expression was clearly upregulated in BC tissues and cell lines. And its expression was positively correlated with both tumor size and TNM stage of BC patients. Further functional experiments elucidated that circ_0042881 deficiency markedly dampened the capacities for proliferation and metastasis both in vitro and in vivo, indicating its tumor-promoting effects in BC. Anti‐sense oligonucleotides (ASOs) are widely used approach for therapeutic RNA editing, which were quickly developing as powerful therapeutics for disease intervention. Several ASO drugs have been approved by the Food and Drug Administration (FDA) for clinical use in diseases [27]. The therapeutic potential of ASOs targeting circ_0042881 in BC should be tested in future research.

Circ_0042881 is derived from exons 2-8 of NF1, and predominantly located at the cytoplasm. Cytoplasmic localization of circ_0042881 motivated us to examine its posttranscriptional regulatory mechanism in BC. Acting as miRNA sponge was a crucial mechanism for those circRNAs located in cytoplasm to function as oncogenic or tumor suppressor regulators [28]. To this end, we first used anti-AGO2 to verify its miRNA binding potential, then analyzed the miRNAs that may associate with circ_0042881 using bioinformatics tools. MiR-217 was screened out to be a circ_0042881 linked miRNA and reported to be dysregulated in various cancer type in previous research. For example, miR-217 has been validated to repress tumor growth and liver metastasis by targeting Kruppel-like factor 5 (KLF5) to activate the mTOR-PI3K-AKT pathway [29]. In pancreatic cancer, miR‐217 was identified to work as a tumor suppressor via directly targeting YWHAG to phosphorylate RAF1 and initiate ERK signaling pathway [30]. Additionally, it imposed a pro-cancer effect in pituitary prolactinoma by obstructing the dickkopf Wnt signaling pathway inhibitor 1 (DKK1) [31]. In our study, we confirmed that miR-217 could interact with circ_0042881 by luciferase assays, and a negative association between miR-217 and circ_0042881 was observed in BC tissues. We demonstrated for the first time that circ_0042881 plays its oncogenic role by acting as a miRNA sponge in BC. The other aspects of circ_0042881 function still await further investigation in BC.

RAS gene is one of the most universally mutated oncogene in human cancers. RAS GTPases cycles between the inactive GDP-bound and active GTP-bound states with the help of guanine nucleotide exchange factors (GEFs) that promote activation, and GTPase-activating proteins (GAPs) that inactivate RAS by catalyzing GTP hydrolysis [32]. In active GTP-bound states, the RAS protein participates in the pathogenesis of tumors by activating downstream signal transduction cascades that support proliferation and survival. SOS1 is one of the most universal and widely expressed GEFs that activates the small GTPase RAS. Here, we verified that SOS1 is a potential functional target of miR-217 by combined RNA-seq and bioinformatic analysis. Dual‐luciferase reporter further corroborated that miR-217 can directly cooperate with SOS1. SOS1 is typically regarded as protumorigenic, which is a crucial binary chemical switch in numerous cellular signaling [33]. Following previous work, SOS1 was post-transcriptionally regulated by miR-483 and promoted BC metastasis by obesity-activated c-Met signaling [17]. The miR-217/SOS1 axis has also been reported in promoting cell viability and cell cycle in acute myelocytic leukemia (AML) cells, which is compatible with our observations [34]. On the basis of prior studies and KEGG and GSEA analysis, we anticipated that MEK/ERK and PI3K/AKT signaling may act as relative pathway in circ_0042881 cancer-promoting process. Western blotting confirmed that circ_0042881 could alter the expression of SOS1, which promoted the phosphorylation levels of AKT and ERK.

Accumulating evidence suggested that trans-acting factors were controlling the production of circRNAs [35]. RBPs can serve as trans-acting elements to promote back-splicing. Dimerization of RBPs, which are attached to certain intron motifs, act as a bridge to bring flanking links closer to each other [36]. In this study, we encountered that NF1 pre-mRNA flanking the circ_0042881 sequence comprised multiple EIF4A3 binding sites, as indicated by CircInteractome database and RIP assay. EIF4A3 is a core component of the exon junction complex (EJC), which could assemble and deposit on canonical sites upstream of exon-exon junctions during splicing [37, 38]. As an RNA binding protein, EIF4A3 can directly combine with pre-mRNA and engage in the back splicing of circRNAs to modulate its expression. Wang et.al firstly confirmed that EIF4A3 involved in circMMP9 cyclization by uniting with its matrix metalloproteinase-9 (MMP-9) mRNA transcript in glioblastoma multiforme [21]. Zheng et.al substantiated EIF4A3-induced circSEPT9 expression in triple-negative BC [39]. There have been further reports of this promoting effect of EIF4A3 on circRNAs biogenesis in other cancers, such as cervical cancer, hepatocellular carcinoma, and colorectal cancer [40–42]. As in the case of our study, circ_0042881 expression was blocked after EIF4A3 silencing. In our BC cohort, EIF4A3 expression was significantly positively associated with circ_0042881 levels.

Taken together, we identified a novel circular RNA, circ_0042881, which was substantially elevated in BC tissues and correlated with TNM stage. Through competitively interacting with miR-217, circ_0042881 can control the expression of SOS1, causing covert into GDP-bound state and activating downstream signal transduction cascades that contribute to the occurrence and development of cancer. In addition, RNA binding protein EIF4A3 promotes its expression through binding to the flanking regions of circ_0042881. Overall, our study identified a new regulatory network that is critical for BC growth and metastasis, and may provide valuable biomarker for BC diagnosis and therapy.

## Materials and methods

### Cell culture

American Type Culture Collection (ATCC, USA) provided the human BC cell lines MDA-MB-231, MCF-7, MDA-MB-415, T47D, ZR-75-1, BT474, AU565, SK-BR-3, HCC1143, CAL51, BT549, and HCC70, as well as the human normal mammary epithelial cell line MCF-10A. All of these cell lines were cultured in DMEM or RPMI 1640 media (Procell Life Science&Technology Co.,Ltd, China) with 10% fetal bovine serum (FBS, VivaCell, China) and 1% penicillin-streptomycin (NCM Biotech, China). All the cells were maintained in a cell culture incubator at 37 °C with 5% CO_2_.

### Patients and samples

Blood samples, tumor tissue and corresponding adjacent non-tumor tissues were acquired from patients who were firstly diagnosed with BC at the First Affiliated Hospital of Zhengzhou University, blood specimens from healthy volunteers were also collected. All patients did not receive any treatment. Adjacent non-tumor specimens were obtained from a standard distance (3 cm) from tumorous tissues of BC patients. Following the resection, the samples were collected and kept in −80 °C freezers until needed. Written informed consent was obtained from each enrolled patient. This study was approved by the Ethics Committee of the First Affiliated Hospital of Zhengzhou University.

### Actinomycin D treatment and RNase R treatment

A total of 5 × 10^5^ MDA-MB-231 and MCF-7 cells were seeded in six-well plates and grown to 70% confluence. 2 μg/mL Actinomycin D (MedChemExpress, NJ, USA) was added to the cells for indicated time points (0, 12, 18, and 24 h) and collected total RNAs. In addition, total RNAs from MDA-MB-231 and MCF-7 was treated with or without 5 U/μg Rnase R (Novoprotein, Shanghai, China) and executed at 37 °C for 15 min. The RT-qPCR was performed to measure the expression of circ_0042881 and its parental gene NF1.

### RNA extraction and real-time quantitative PCR (RT-qPCR)

The total RNA of cells and tissues was extracted using Trizol (Takara, Japan). Plasma RNAs were extracted from 2 mL of plasma using TRI Blood/Liquid sample total RNA extraction kit (Genenode, China) according to the manufacturer’s guidelines. The PrimeScript™ RT Master Mix (Takara, Japan) was utilized for cDNA synthesis. The miRNA reverse transcription was carried on by using Mir-X^TM^ miRNA First-Strand Synthesis Kit (Takara, Japan). The RT-qPCR was conducted to measure RNA expression using Hieff qPCR SYBR Green Master Mix (YEASEN, China). The relative gene expression levels were quantified using the 2^–ΔΔCt^ method. GAPDH and U6 were used as reference genes. The primers used for qPCR are presented in Table S2.

### RNA interference and lentivirus-mediated infection

Small interfering RNA (siRNA) targeting circ_0042881, EIF4A3, SOS1, and their relative controls, as well as miR-217 mimic, mimic negative control, miR-217 inhibitor, and inhibitor negative control, were purchased from RiboBio (Guangzhou, China). Transfection was carried out using the RiboFECT CP Transfection Kit (Guangzhou, China). The overexpression of circ_0042881 or SOS1 was constructed using the lentivirus-based vectors by GeneChem (Shanghai, China). After transfection, stably transfected cells were filtered at a concentration of 6 μg/mL puromycin (TargetMol, USA). The transfection efficiency was measured by RT-qPCR or western blotting. The sequence of siRNA was listed in Table S3.

### Cell proliferation assays

CCK-8 (NCM Biotech, Suzhou, China) assay and EdU (APExBIO, Houston, USA) assay were conducted following the guidelines of the manufacturer to identify cell proliferation. For CCK-8 assay, 10 µL of CCK-8 reagent mixed with 90 µL of medium was added to each well, then the plate was incubated at 37 °C for 2 h. Afterwards, the absorbance at the wavelength of 450 nm (OD value) was measured by microplate assay. For EdU assay, cells were stained with EdU reagent for 2 h and fixed with 4% paraformaldehyde for 30 min. Fluorescence signal was observed under an inverted fluorescence microscope. The proportion of EdU-positive cells was analyzed by Image J.

### Cell migration assays

Cell migration was analyzed by transwell and wound-healing assays. For the transwell assay, cells after transfection were seeded in the upper chamber at 2 × 10^4^ cells, while 500 μL medium blended with 10% FBS was added to the lower side. After 24 h incubation, transwell chambers were fixed with 4% paraformaldehyde and stained with crystal violet. After wiping the non-migratory cells from upper chamber, the cells in the lower chamber were counted under an inverted microscope. For the wound‐healing assay, after corresponding treatments, cells were cultured in 6-well plates until the confluence reached 80 % and scratched with a 200 μL pipette tip. Photographs were taken under an inversed microscope at 0/48 h after cell scratch. The proportion of wound-healing area was analyzed by Image J.

### Animal models

All animal experimental protocols were conducted in accordance compliance with the Institutional Animal Care and Use Committee of the First Affiliated Hospital of Zhengzhou University. Six-week-old female NOD/SCID mice were purchased from Vital River Laboratory Animal Technology Co. Ltd (Beijing, China). Mice were randomly assigned to each group. For orthotopic BC mouse models, single-cell suspensions (5 × 10^6^ MDA-MB-231 cells with indicated vectors) were inoculated into the fourth mammary fat pad of NOD/SCID mice in volume of 100 μL sterile PBS and matrigel (1:1). Mice were checked every 5 days for examining for mammary tumor development and tumor were calculated as previously described [43]. Maximum tumor diameters were not exceeded 20 mm at the endpoint. For experimental lung metastasis assays, 2 × 10^6^ control or circ_0042881 overexpressing MDA-MB-231 cells were intravenously injected to the tail veins of NOD/SCID mice (six mice per group). After 7 weeks of observation, mice were euthanized, and the lung tissues were harvested for pathological analysis.

### Statistical analysis

The data of all experiments are presented as means ± standard deviation (SD). Statistical analyses were conducted using SPSS 20.0 (Chicago, USA) and GraphPad Prism 9.0 (La Jolla, CA, USA). Statistical expression differences between two groups were determined using the paired or unpaired Student’s t-test. Pearson correlation analysis was used to analyze the correlations among circ_0042881, miR-217, and SOS1. Clinicopathologic parameters and immunohistochemistry results were tested using the Chi-square test or Fisher’s exact test, as appropriate. *P* < 0.05 was considered statistically significant.

Other methods are provided as supplementary files.

## Supplementary information

Supplementary methods and figures

Reproducibility checklist

Authorship Change Approval

Original western blots

## Data Availability

All relevant data are available from the corresponding authors upon request. The RNA-seq data have been deposited in the NCBI Gene Expression Omnibus under accession unmber GSE234870.
