# Supplementary material for: EIF4A3-mediated circ_0042881 activates the RAS pathway via miR-217/SOS1 axis to facilitate breast cancer progression
Source: Cell Death Dis. 2023 Aug 25;14(8):559. doi: 10.1038/s41419-023-06085-4 (PMC10457341; doi:10.1038/s41419-023-06085-4)
Supplement: Supplementary file 1 — Supplementary methods and figures [file 41419_2023_6085_MOESM1_ESM.pdf]

Supplementary Information

EIF4A3-mediated circ_0042881 activates the RAS pathway via miR-217/SOS1 axis to facilitate breast cancer progression

Chenxi Ju, Mingxia Zhou, Dan Du, Chang Wang, Jieqiong Yao, Hongle Li, Yang Luo, Fucheng He, Jing He

This PDF file includes:

Supplementary Materials and Methods

Figure S1-S8

Table S1-S3

Nucleocytoplasmic fractionation

The separation of cytoplasmic and nuclear RNA in MDA-MB-231 and MCF-7 was conducted using NE-PER™ Nuclear and Cytoplasmic Extraction Reagents (Thermo Fisher, USA). RNA samples extracted from the fractions of cytoplasmic and nuclear were used for RT-qPCR. U6 was used as the nucleus reference and GAPDH was used as the cytoplasmic reference.

Western blotting

Radio immunoprecipitation assay (RIPA) Lysis Buffer and 1% PMSF (Beyotime, China) resuspended cell pellets and incubated on the ice for 30 min and vortexed per 10 min. Cell lysate was centrifuged at 12000 g for 5 min and the supernatant was retained. Total protein concentration was quantified using a BCA kit (Epizyme, China). The equal protein amounts with different molecular weight were subjected into sodium dodecyl sulfate-polyacrylamide gel electrophoresis (SDS-PAGE) to proceed separated and then blotted onto the polyvinylidene difluoride (PVDF) membranes. After blocking with 5% skim milk powder, the membranes were incubated with primary antibodies at 4 °C overnight and followed incubation with the secondary antibodies for 2 h at room temperature. The membranes were visualized using ECL chemiluminescent reagent (Epizyme, China). Antibodies used for western blotting and their dilutions were as follows: anti-SOS1 (55041-1-AP, 1:1000, Proteintech, China), anti-EIF4A3 (17504-1-AP, 1:1000, Proteintech, China), anti-β-actin (55041-1-AP, 1:5000, Proteintech, China), anti-AKT (10176-2-AP, 1:1000, Proteintech, China), anti-p-AKT (66444-1-Ig,

1:1000, Proteintech, China), anti-ERK (16443-1-AP, 1:1000, Proteintech, China), anti-p-ERK (9101, 1:1000, Cell Signaling Technology, USA).

RNA-seq

The total RNA was collected using Trizol (Takara, Japan). RNA-seq libraries from 2 μg total RNA were prepared using KCTM Stranded mRNA Library Prep Kit for Illumina® (Wuhan, China), according to the manufacturer's instructions. PCR products corresponding to 200-500 bps were enriched, quantified and finally conducted by DNBSEQ-T7 sequencer with PE150 model. The RNA-seq data have been deposited in the NCBI Gene Expression Omnibus under accession number GSE234870.

RNA fluorescence in situ hybridization (FISH)

Cy3-labeled probe specific to circ_0042881 (RioBio, Guangzhou, China) was used to observe the cellular localization of circ_0042881 in MCF-7. Cells were fixed with 4% paraformaldehyde and then incubated with probes overnight. The nuclei were counterstained with DAPI.

Colony formation assay

The transfected cells were collected and seeded at a density of 1000 cells per well in 6-well plates with culture medium containing 10% FBS, and cultured for 2 weeks until visible colonies were observed. The cells were fixed with 4% paraformaldehyde and stained with 0.1% crystal violet. The visible clone number was counted to indicate the

ability of cell proliferation.

Luciferase reporter assay

The binding relationship among circ_0042881, miR-217, and SOS1 was confirmed by a luciferase assay. The wild type sequences and corresponding mutant sequences that bind to miR-217 in circ_0042881 and SOS1 3'UTR was cloned and inserted into psiCHECK-2 plasmid (Hanbio, China) containing the firefly and Renilla luciferase reporter genes. MDA-MB-231 and MCF-7 were co-transfected with dual-luciferase reporter vector and the mimic or NC of miR-217. The activity of firefly and Renilla luciferase in the cells was measured with a Dual-Luciferase Assay Kit (Beyotime, China).

RNA immunoprecipitation (RIP) assay

RIP assay was carried out using Magna RIP™ RNA-Binding Protein Immunoprecipitation Kit (Millipore, MA, USA) according to the manufacturer's instructions. RIP lysis buffer containing RNase and protease inhibitors was used to lyse transfected cells. The cell lysates were incubated with antibody with magnetic beads at 4 °C overnight. Then, the immunoprecipitates were washed with lysis buffer and then incubated with proteinase K at 55 °C for 30 minutes with shaking. Finally, the elution was added with the immune precipitated RNAs for subsequent RNA extraction and followed RT-qPCR analysis.

Immunohistochemistry stain (IHC)

The obtained BC tissues were fixed in paraffin and cut into 4-μm-thick sections. These sections were then deparaffinized and immersed in H_2O_2 at room temperature for 20 min. Then tissues were exposed to treatment with goat serum for 30 min to eliminate non-specific staining. Sections were subsequently incubated with the primary antibodies anti-SOS1 (55041-1-AP, 1:200, Proteintech, China), anti-EIF4A3 (17504-1-AP, 1:1000, Proteintech, China) and kept at 4°C overnight. Unbound antibody was removed by washing in PBS. This was followed by incubation with the biotinylated anti-IgG secondary antibody and horseradish peroxidase-labeled streptavidin. The immunohistochemical results were scored by multiplying the staining intensity by the proportion of positive cancer cells. The percentage of positive cells was graded as follows: 0 (<10 %), 1 (10-25 %), 2 (25-50 %) and 3 (50-75 %), and 4 (>75 %). The intensity of stain was scaled as: 0 (negative), 1 (weak), 2 (moderate), and 3 (strong).

Supplementary Figures and Figure legends

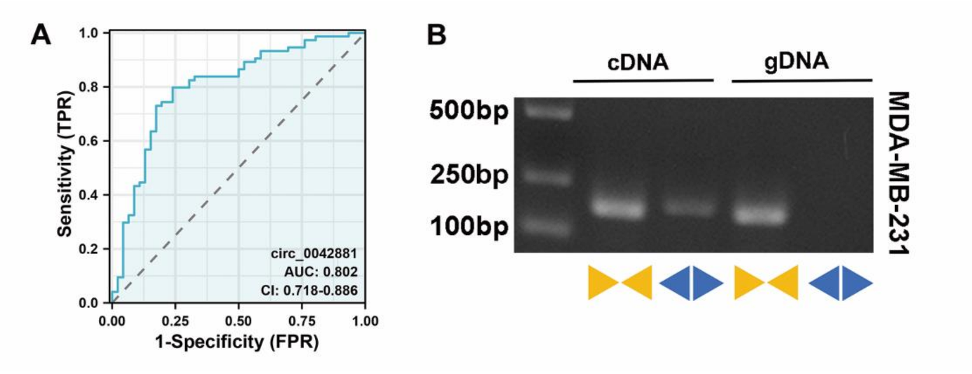

Fig. S1. Circ_0042881 is highly expressed in breast cancer. (A) The ROC curve was conducted to estimate the diagnostic value of circ_0042881 in plasma as a biomarker for BC. (B) PCR with convergent and divergent primers and agarose gel electrophoresis to verify the circular structure of circ_0042881 in MDA-MB-231; divergent and convergent primers are indicated by the direction of the arrow.

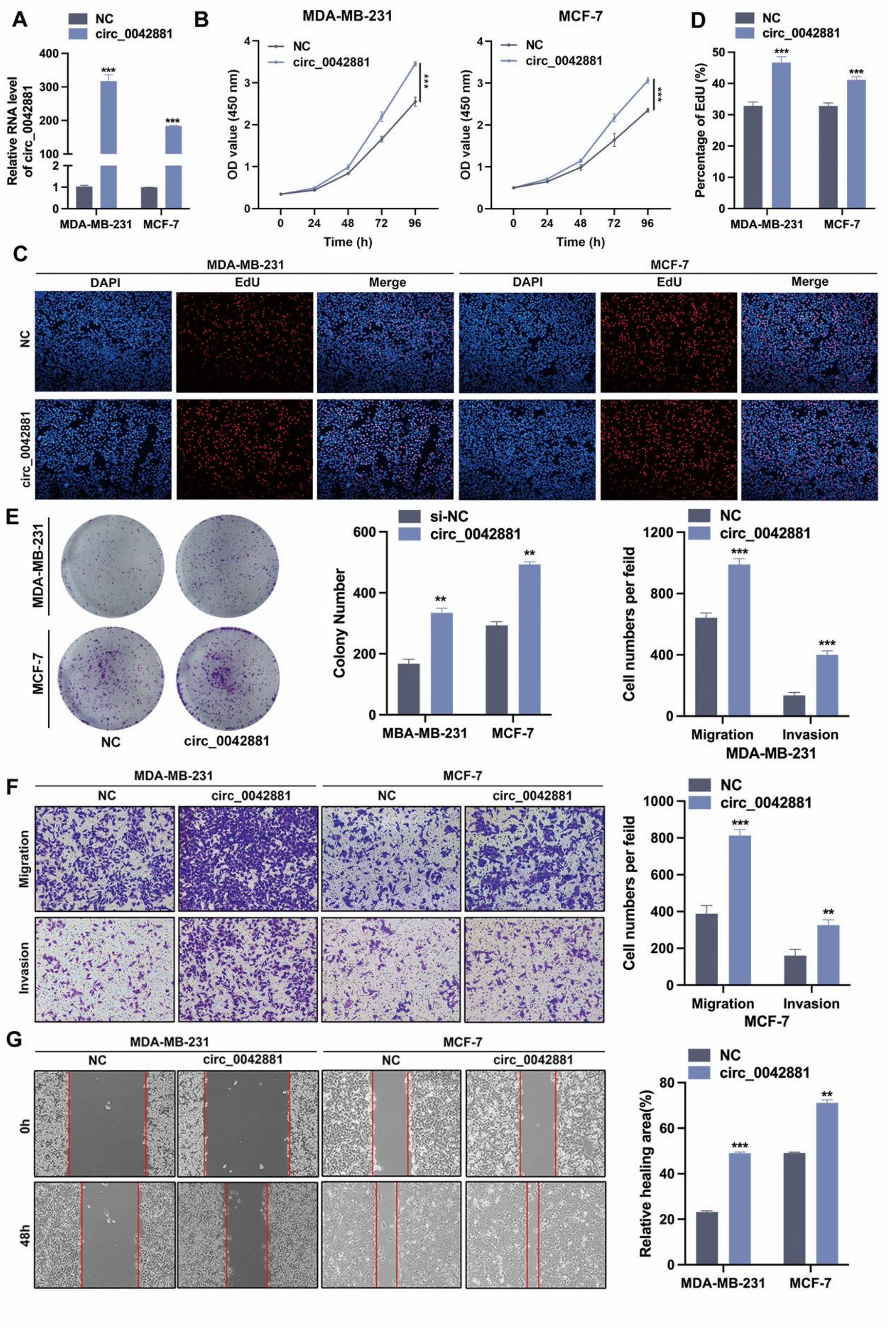

Fig. S2. Overexpression of circ_0042881 promotes BC progression. (A) RT-qPCR analysis of the relative circ_0042881 expression after circ_0042881 overexpression in

MDA-MB-231 and MCF-7. (B-E) The effect of circ_0042881 overexpression on proliferation in MDA-MB-231 and MCF-7 were tested by CCK-8, EdU and colony formation assays. (F) Transwell assays were performed to analyze cell migration and invasion ability. The migration and invasion cell numbers were shown in right panel. (G) Wound healing assays were used to evaluate the cell migration ability. The data are presented as mean ± SD. **$P < 0.01$, ***$P < 0.001$.

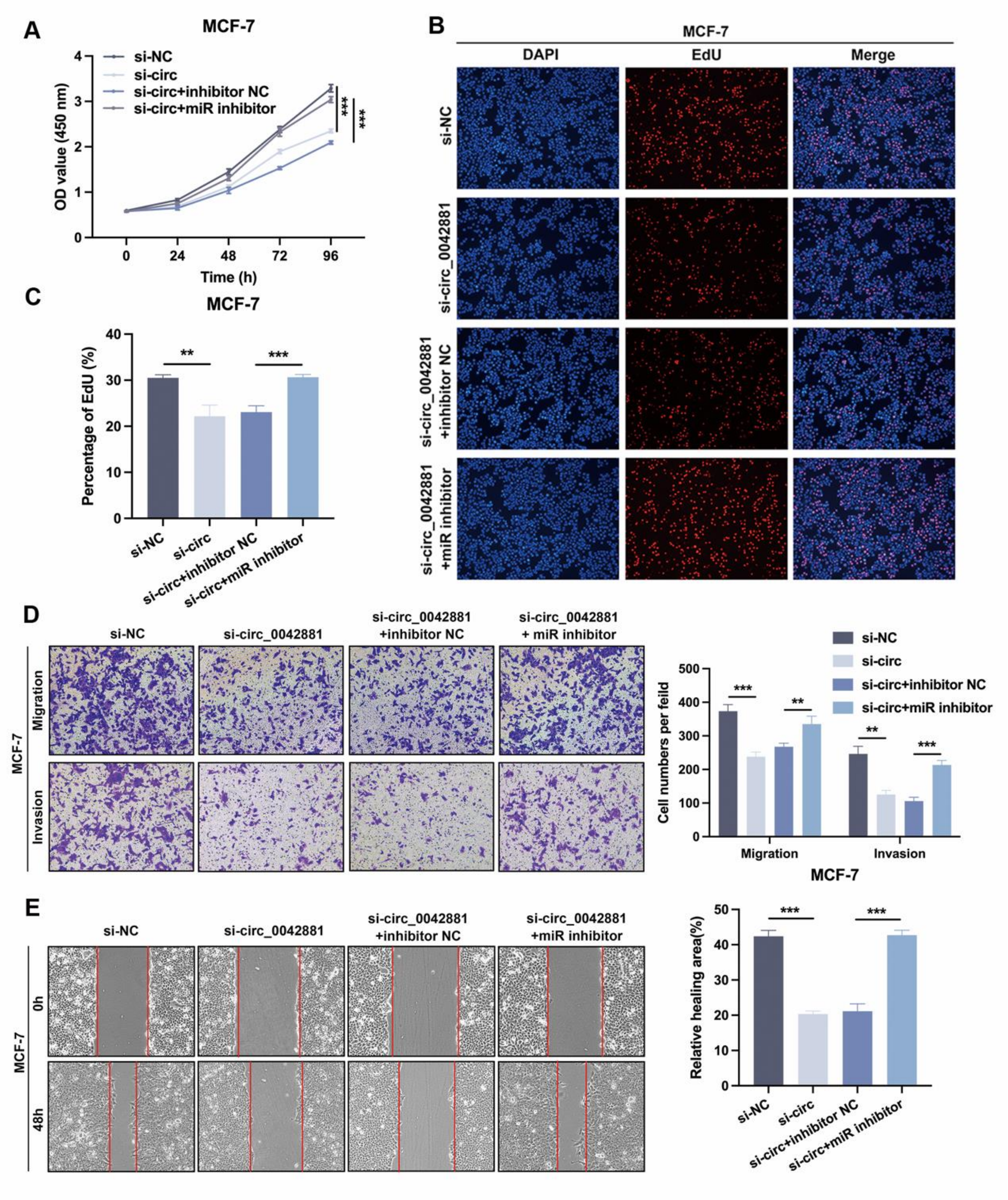

Fig. S3. Circ_0042881 promoted BC progression by inhibiting miR-217. (A-C) CCK-8, EdU assays were performed to assess cell proliferation ability of each group. (D) The capacity of cell migration and invasion of each group was determined by transwell assays. (E) The effect of si-circ_0042881 and miR-217 inhibitor on migration was examined by wound healing assays. The data are presented as mean ± SD. **P < 0.01, ***P < 0.001.

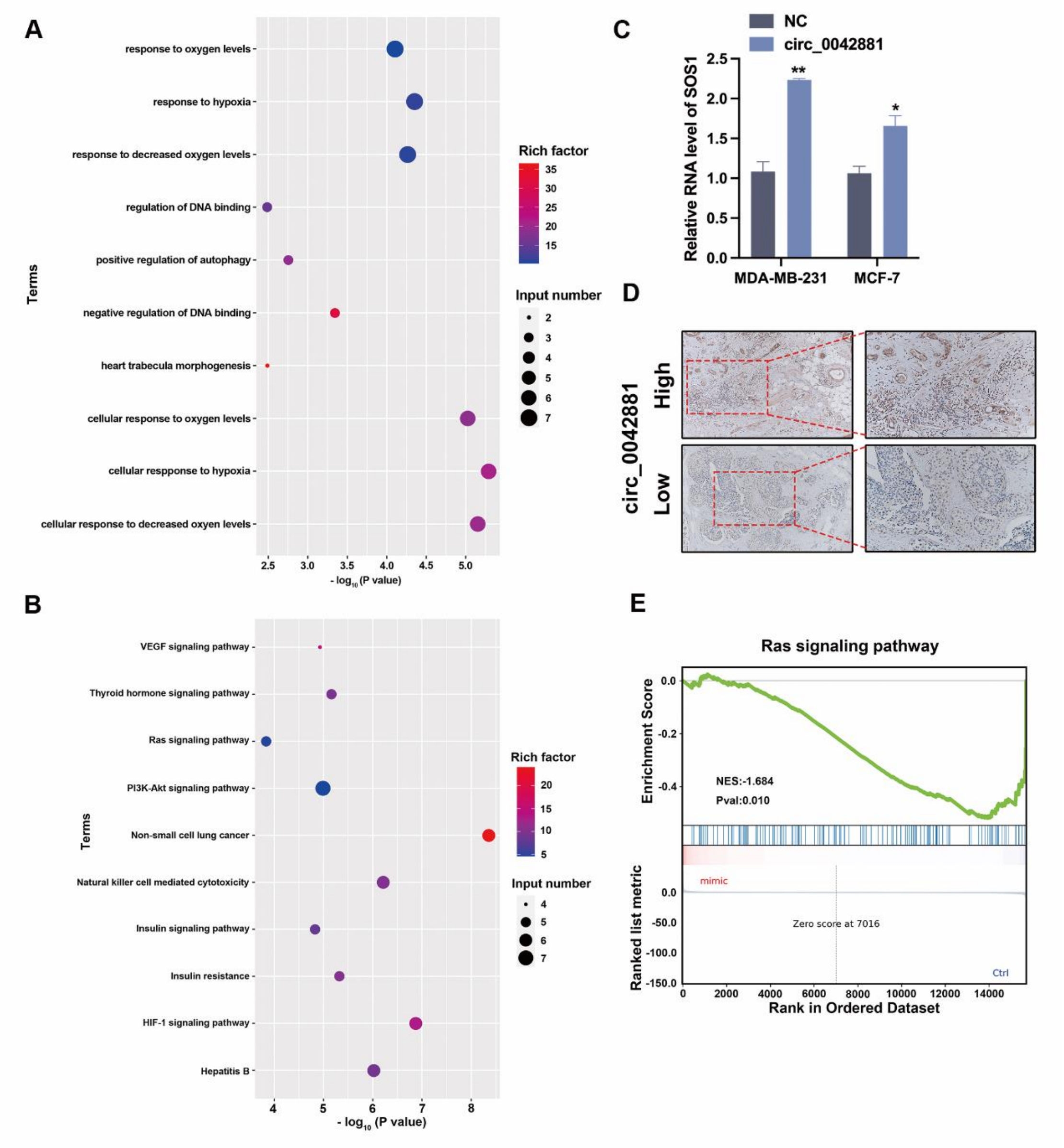

Fig. S4. SOS1 is targeted by miR-217. (A) GO function analysis of differentially expressed mRNAs after transfecting miR-217 mimic. (B) KEGG pathway enrichment analysis of differentially expressed mRNAs after transfecting miR-217 mimic. (C) The relative expression of SOS1 was detected after circ_0042881 overexpression using RT-qPCR. (D) IHC results showed that higher circ_0042881 expression was related to higher expression of EIF4A3 protein in BC tissues. (E) GSEA analysis showed a significant enrichment in RAS signaling pathway. The data are presented as mean ± SD. $*P < 0.05$, $**P < 0.01$.

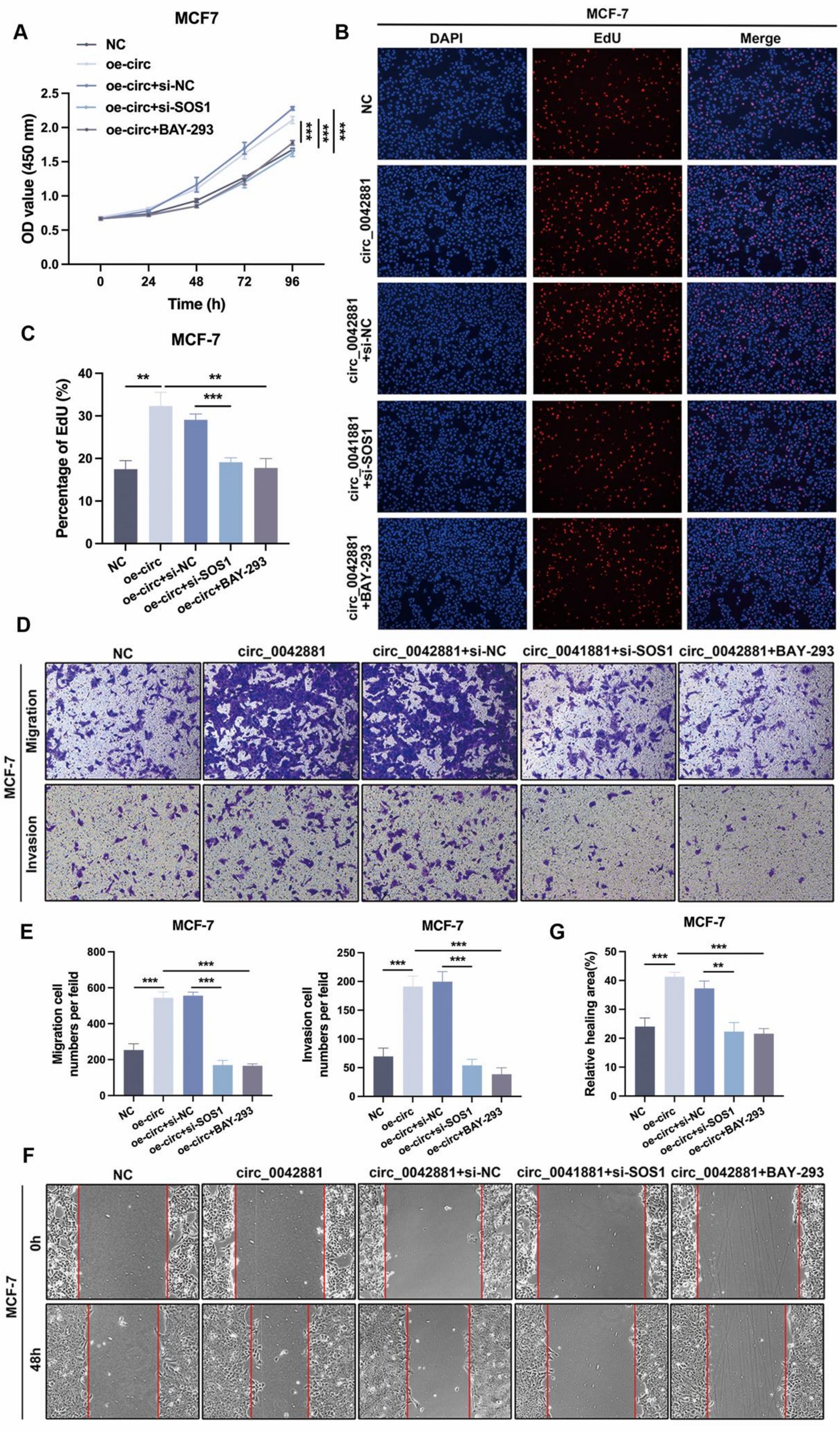
A
MCF7
NC
oe-circ
oe-circ+si-NC
oe-circ+si-SOS1
oe-circ+BAY-293
OD value (450 nm)
Time (h)
***
B
MCF-7
DAPI
EdU
Merge
NC
circ_0042881
circ_0042881+si-NC
circ_0041881+si-SOS1
circ_0042881+BAY-293
C
MCF-7
Percentage of EdU (%)
D
NC
circ_0042881
circ_0042881+si-NC
circ_0041881+si-SOS1
circ_0042881+BAY-293
Migration
Invasion
MCF-7
E
MCF-7
Migration cell numbers per feild
Invasion cell numbers per feild
G
MCF-7
Relative healing area(%)
F
NC
circ_0042881
circ_0042881+si-NC
circ_0041881+si-SOS1
circ_0042881+BAY-293
0h
48h
MCF-7

Fig. S5. Circ_0042881 promotes proliferation and metastasis in a SOS1-dependent manner. (A-C) The proliferation ability of MCF-7 was detected by CCK-8 and EdU assays treated as indicated. (D-E) The migration and invasion ability of MCF-7 was detected by transwell assays treated as indicated. (F, G) Wound healing assays were performed to detect the migration ability of MCF-7 cells treated as indicated. The data are presented as mean ± SD. **$P < 0.01$, ***$P < 0.001$.

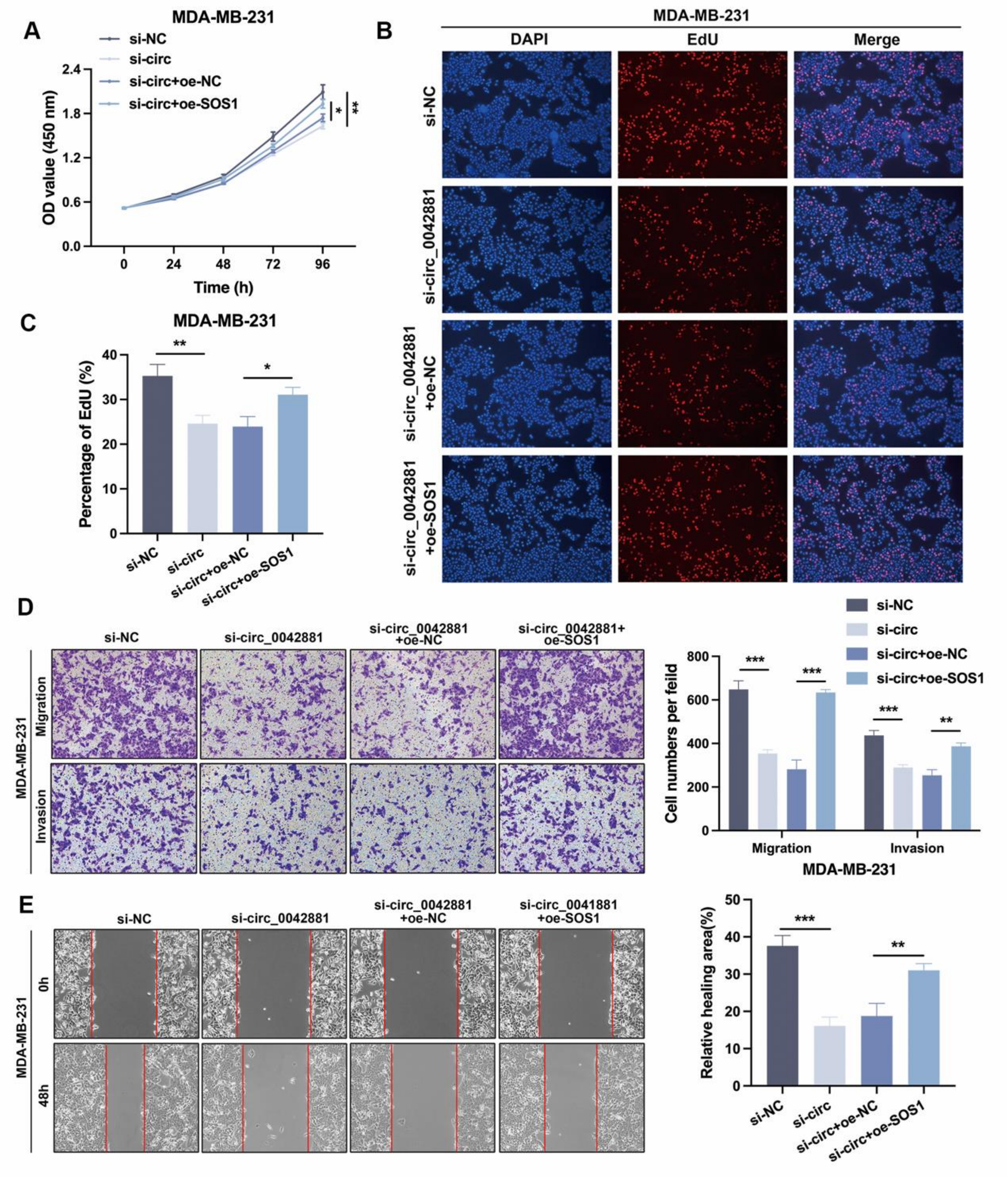

Fig. S6. SOS1 could rescue the suppressive effect of circ_0042881 knockdown on malignant phenotypes of MDA-MB-231. (A-C) CCK-8, EdU assays were performed to assess cell proliferation ability of each group. (D) The capacity of cell migration and invasion of each group was determined by transwell assays. (E) The effect of si-circ_0042881 and oe-SOS1 on migration was examined by wound healing assays. The data are presented as mean ± SD. **P* < 0.05, **P < 0.01, ***P < 0.001.

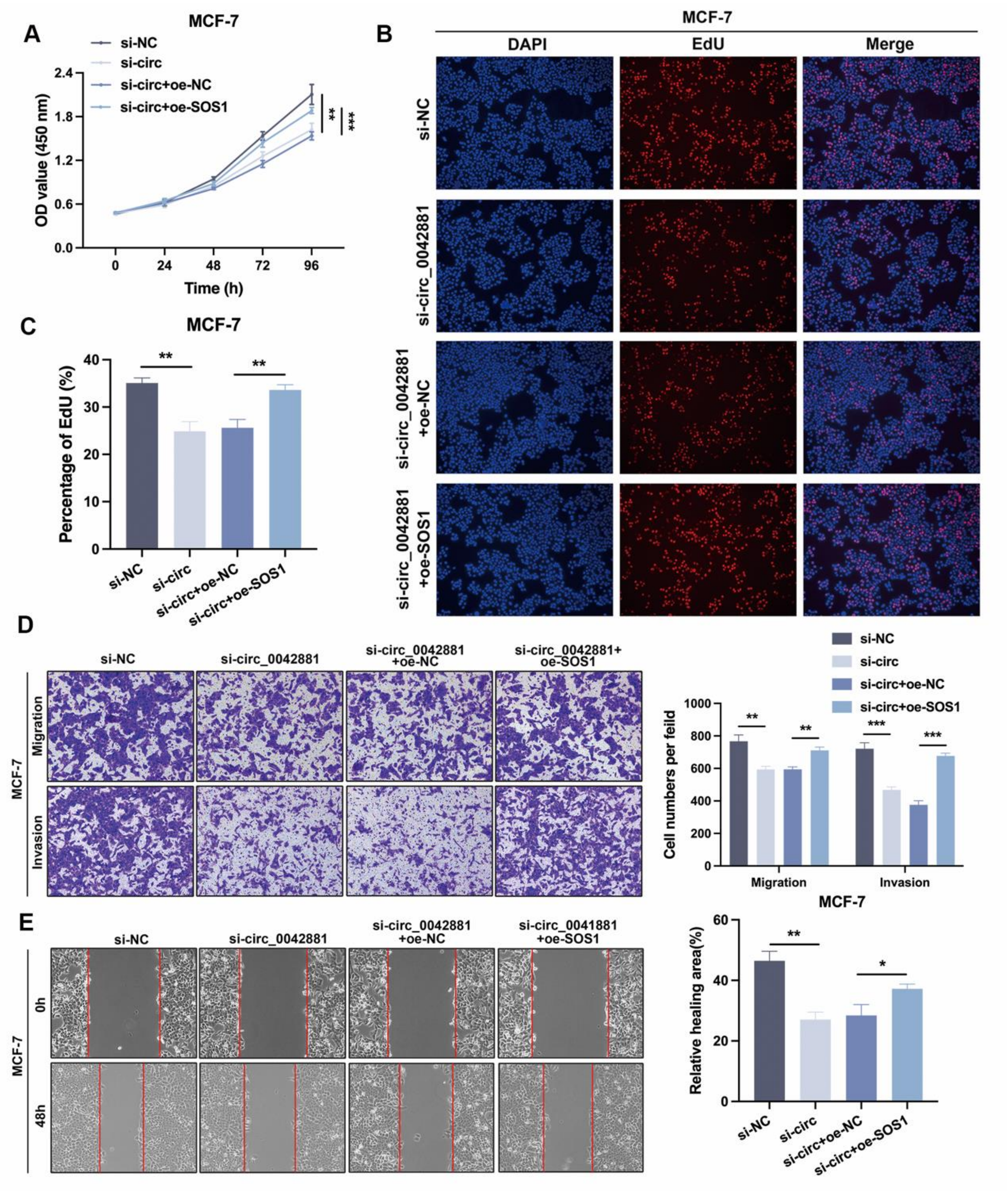

Fig. S7. SOS1 could rescue the suppressive effect of circ_0042881 knockdown on malignant phenotypes of MCF-7. (A-C) CCK-8, EdU assays were performed to assess cell proliferation ability of each group. (D) The capacity of cell migration and invasion of each group was determined by transwell assays. (E) The effect of si-circ_0042881 and oe-SOS1 on migration was examined by wound healing assays. The data are presented as mean ± SD. *$P < 0.05$, **$P < 0.01$, ***$P < 0.001$.

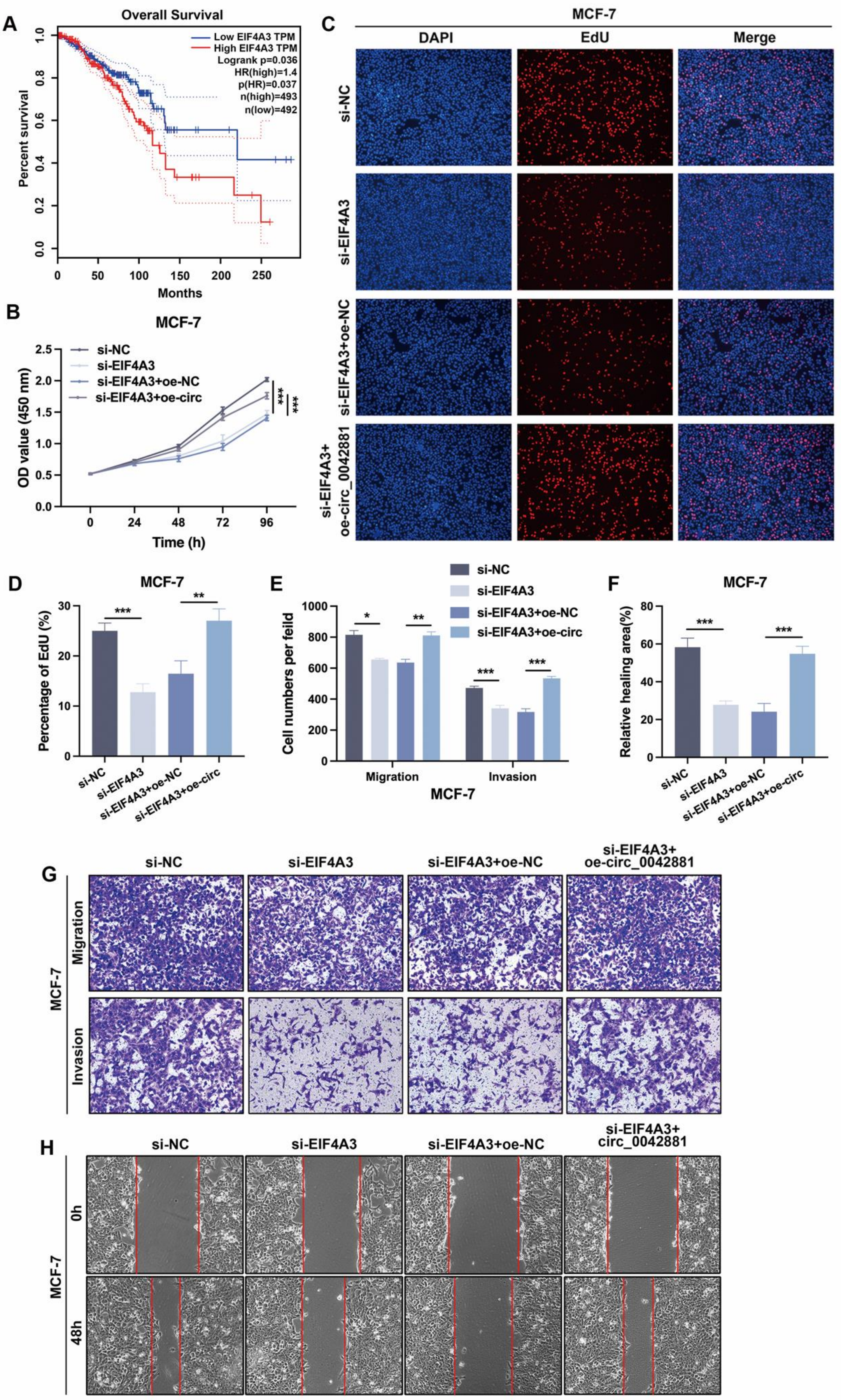

A
Overall Survival
Low EIF4A3 TPM
High EIF4A3 TPM
Logrank p=0.036
HR(high)=1.4
p(HR)=0.037
n(high)=493
n(low)=492
Percent survival
Months
B
MCF-7
si-NC
si-EIF4A3
si-EIF4A3+oe-NC
si-EIF4A3+oe-circ
OD value (450 nm)
Time (h)
C
MCF-7
DAPI
EdU
Merge
si-NC
si-EIF4A3
si-EIF4A3+oe-NC
si-EIF4A3+ oe-circ_0042881
D
MCF-7
Percentage of EdU (%)
E
Cell numbers per feild
Migration
Invasion
MCF-7
F
MCF-7
Relative healing area(%)
G
si-NC
si-EIF4A3
si-EIF4A3+oe-NC
si-EIF4A3+ oe-circ_0042881
MCF-7
Migration
Invasion
H
si-NC
si-EIF4A3
si-EIF4A3+oe-NC
si-EIF4A3+ circ_0042881
MCF-7
0h
48h

Fig. S8. RNA binding protein EIF4A3 regulates the expression of circ_0042881. (A) The relationship between EIF4A3 expression and overall survival of BC patients was predicted from GEPIA. (B-D) The proliferation ability of MCF-7 was detected by CCK-8 and EdU assays treated as indicated. (D-E) The migration and invasion ability of MCF-7 was detected by transwell and wound healing assays treated as indicated. The data are presented as mean ± SD. $^{**}P < 0.01$, $^{***}P < 0.001$.

Table S1. The correlation between circ_0042881 and BC clinicopathologic features.

	Circ_0042881 expression		*P*-value
	High (n=28)	Low (n=28)	
**Tumor size**			
T_1	2	8	
T_2	21	19	**0.041***
T_3	5	1	
**Lymph node metastasis**			
N_0	16	16	
N_1	7	5	0.717
$N_{2\text{-}3}$	5	7	
**TNM stage**			
I	1	6	**0.043***
II + III + IV	27	22	
**ER status**			
Positive (≥ 10%)	16	15	0.788
Negative (< 10%)	12	13	
**PR status**			
Positive (≥ 10%)	12	14	0.592
Negative (< 10%)	16	14	
**Ki67 index**			
Low (≤15%)	4	4	1
High (>15%)	24	24	

Table S2. Sequences of primers used for RT-qPCR in this study.

Name	Sequence
Circ_0042881 Forward	ATGAAAACAACATGAATAAGC
Circ_0042881 Reverse	TTTTCAGCAGCTTCTCCAAATA
hsa-miR-545-3p Forward	CGCGAGCGCTCAGTAAATGTTTATTAG
hsa-miR-382-5p Forward	CTGAAGTTGTTCGTGGTGGATTCG
hsa-miR-217 Forward	GCTACTGCATCAGGAACTGATTGGA
hsa-miR-338-5p Forward	GCGTCCAGCATCAGTGATTTTGTTG
Circ_0042881 convergent primer Forward	GCTGGTCAAACAGTTGCTGC
Circ_0042881 convergent primer Reverse	TTGAAGTTGTTGCAGCTGAGAG
EIF4A3 Forward	TGGCTCCCACAAGAGAGTTG
EIF4A3 Reverse	GCACTGGACATTCATGTAGTCA
SOS1 Forward	GAGTGAATCTGCATGTCGGTT
SOS1 Reverse	CTCTCATGTTTGGCTCCTACAC
GAPDH Forward	AAGGTGAAGGTCGGAGTCA
GAPDH Reverse	GGAAGATGGTGATGGGATTT
UBL3 Forward	AGTAATGTCCCGGCGGATATG
UBL3 Reverse	TCAGAAGCAGAATCGTTAGGAGA
SPOPL Forward	TACTGGTCCCATAGCAGAAAGC
SPOPL Reverse	CCCATTTCCTCTCGACAAAAACT
ICK Forward	AGCTCAACCATGCCAATGTAG
ICK Reverse	AGTCTCGATGAAAGAAGCCGT
INSIG2 Forward	TATACCCCTGCATTGACAGACA
INSIG2 Reverse	CTACACACCGCATTACACTGG
U6 Forward	CTCGCTTCGGCAGCACA
U6 Reverse	AACGCTTCACGAATTTGCGT
NF1 Forward	CGAATCATCACCAATTCCGCA

NF1 Reverse	CCACAACCTTGCACTGCTTTAT

Table S3. Sequences of siRNA and shRNA used in this study.

Gene name	Probe (5’-3’)
si-circ_0042881#1	CAACATGAATAAGCTTCCA
si-circ_0042881#2	ACATGAATAAGCTTCCAAT
si-EIF4A3	GCTGGATTACGGACAGCAT
si-SOS1	GCCTTACTGTTTACGAGTA
