# Supplementary material for: EIF4A3-mediated circ_0042881 activates the RAS pathway via miR-217/SOS1 axis to facilitate breast cancer progression
Source: Cell Death Dis. 2023 Aug 25;14(8):559. doi: 10.1038/s41419-023-06085-4 (PMC10457341; doi:10.1038/s41419-023-06085-4)
Supplement: Supplementary file 3 — Authorship Change Approval [file 41419_2023_6085_MOESM3_ESM.pdf]

Chenxi Ju

Re: Confirmation of changes in Authorship 精简信息

发件人: . <1289474974@qq.com>
时 间: 2023年08月16日 23:09:08 (星期三)
收件人: 贺付成 <hefucheng@zzu.edu.cn>

Yes， I agree to the author list being changed.

——Original——

From: "贺付成"<hefucheng@zzu.edu.cn>
Date: Wed, Aug 16, 2023 23:06 PM
To: "居晨曦"<1289474974@qq.com>;"mingxiazhou0906"<mingxiazhou0906@163.com>;"3417631572"<3417631572@qq.com>;"汪唱"<1457572286@qq.com>;"1104417059"<1104417059@qq.com>;"llhl73"<llhl73@163.com>;"luoy"<luoy@cqu.edu.cn>;"hejing92921"<hejing92921@163.com>;
Subject: Confirmation of changes in Authorship

Dear Co-authors,

As mentioned before, our manuscript (CDDIS-23-1052) has been accepted in principle by Cell Death & Disease. We are preparing the very final revision. We changed the author list by adding Yang Luo who contribution in the revisions. Please check below.
The current author list is — Chenxi Ju[1,7], Mingxia Zhou[2,7], Dan Du[1,7], Chang Wang[1], Jieqiong Yao[3], Hongle Li[4], Yang Luo[1,5*], Fucheng He[1*], Jing He[6*]

If you agree to the authorship arrangement, please confirm the change of the authorship by replying this email with "Yes, I agree to the author list being changed".

Thank you very much.

Fucheng He

Mingxia Zhou

Re: Confirmation of changes in Authorship 精简信息

发件人: 周明霞 <mingxiazhou0906@163.com>
时 间: 2023年08月16日 23:10:45 (星期三)
收件人: 贺付成 <hefucheng@zzu.edu.cn>

Yes, I agree to the author list being changed.

---- Replied Message ----

Dan Du

Re: Confirmation of changes in Authorship 精简信息

发件人: 郑州大学 杜丹 <3417631572@qq.com>
时 间: 2023年08月16日 23:10:06 (星期三)
收件人: 贺付成 <hefucheng@zzu.edu.cn>

Yes, I agree to the author list being changed.

Chang Wang

Re: Confirmation of changes in Authorship 精简信息

发件人: 汪唱 <1457572286@qq.com>
时 间: 2023年08月17日 00:07:35 (星期四)
收件人: 贺付成 <hefucheng@zzu.edu.cn>

Yes, I agree to the author list being changed.

Jieqiong Yao

Re: Confirmation of changes in Authorship 精简信息

发件人: YJQ <1104417059@qq.com>
时 间: 2023年08月16日 23:08:45 (星期三)
收件人: 贺付成 <hefucheng@zzu.edu.cn>

Yes, I agree to the author list being changed.

Hongle Li

回复：Confirmation of changes in Authorship 精简信息

发件人： 李红乐 <llhl73@163.com>
时　间： 2023年08月17日 07:43:30 (星期四)
收件人： hefucheng <hefucheng@zzu.edu.cn>

Yes, I agree to the author list being changed.

Yang Luo

Re: Confirmation of changes in Authorship 精简信息

发件人： luoy@cqu... <luoy@cqu.edu.cn>
时　间： 2023年08月16日 23:27:44 (星期三)
收件人： 贺付成 <hefucheng@zzu.edu.cn>

Yes, thanks.
Yang

Fucheng He

Re: Confirmation of changes in Authorship 精简信息

发件人： 贺付成 <hefucheng@zzu.edu.cn>
时　间： 2023年08月16日 23:16:04 (星期三)
收件人： 贺付成 <hefucheng@zzu.edu.cn>

Yes, I agree to the author list being changed.

Jing He

Re:Confirmation of changes in Authorship 精简信息

发件人： 贺靖 <hejing92921@163.com>
时　间： 2023年08月16日 23:08:05 (星期三)
收件人： 贺付成 <hefucheng@zzu.edu.cn>

Yes, I agree to the author list being changed.
