# Supplementary material for: EIF4A3-mediated circ_0042881 activates the RAS pathway via miR-217/SOS1 axis to facilitate breast cancer progression
Source: Cell Death Dis. 2023 Aug 25;14(8):559. doi: 10.1038/s41419-023-06085-4 (PMC10457341; doi:10.1038/s41419-023-06085-4)
Supplement: Supplementary file 4 — Original western blots [file 41419_2023_6085_MOESM4_ESM.docx]

**Fig.5H AKT**


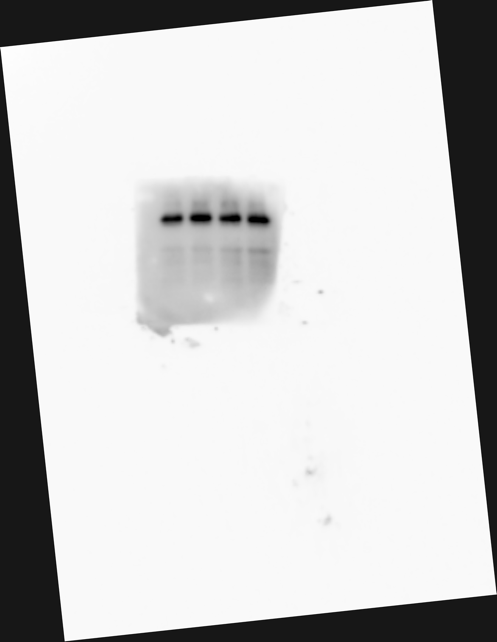


**Fig.5H ERK**


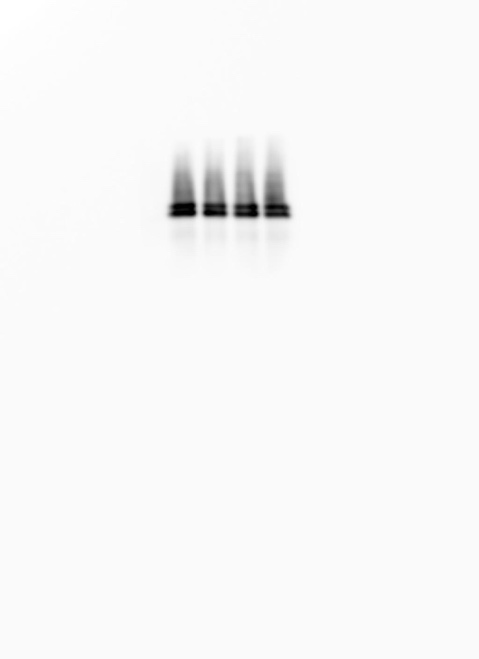


**Fig.5H p-ERK**


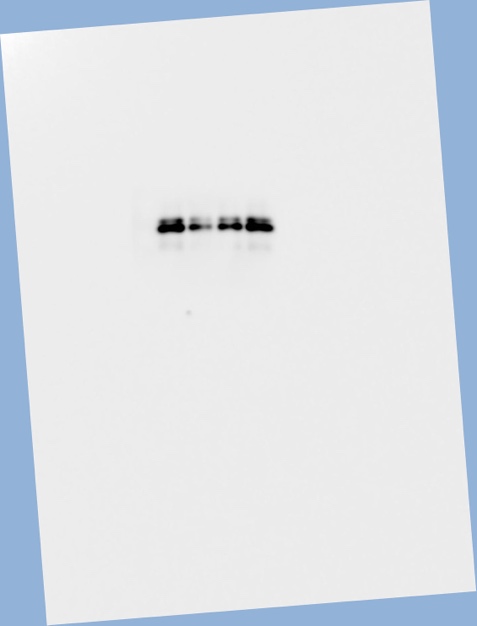


**Fig.5H p-AKT**


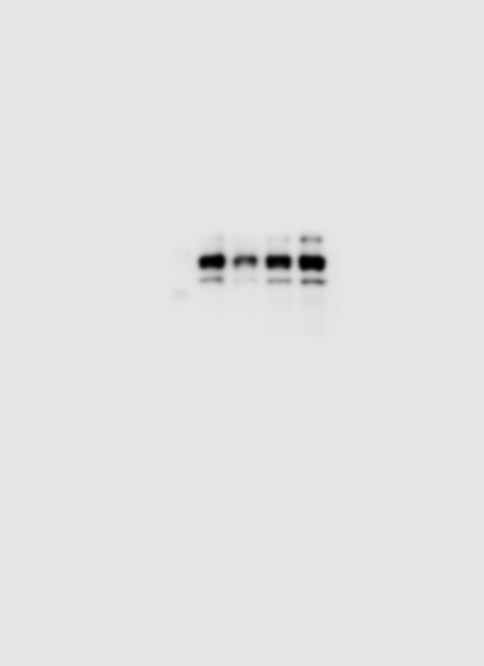


**Fig.5H SOS1**


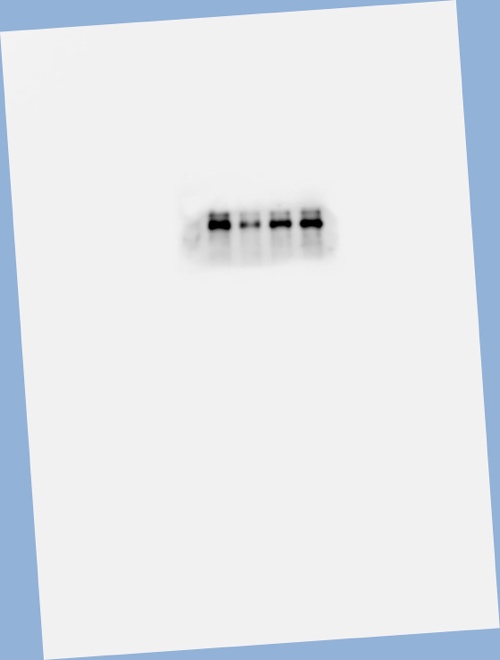


**Fig.5H β-Actin**


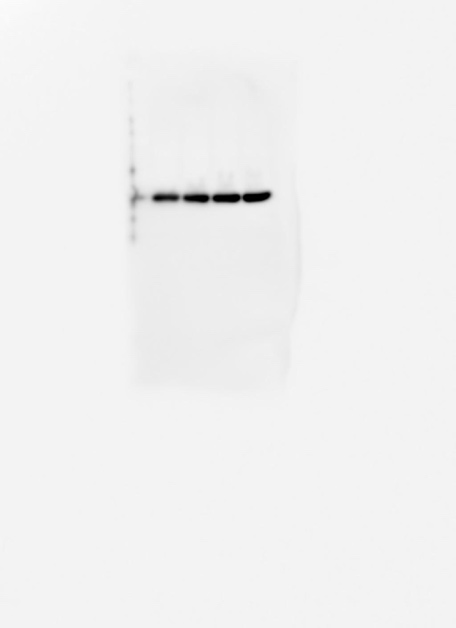


**Fig.5I AKT**

**
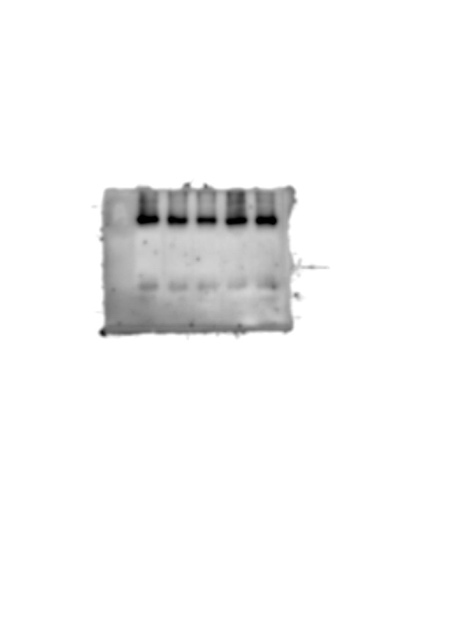
**

**Fig.5I ERK**

**
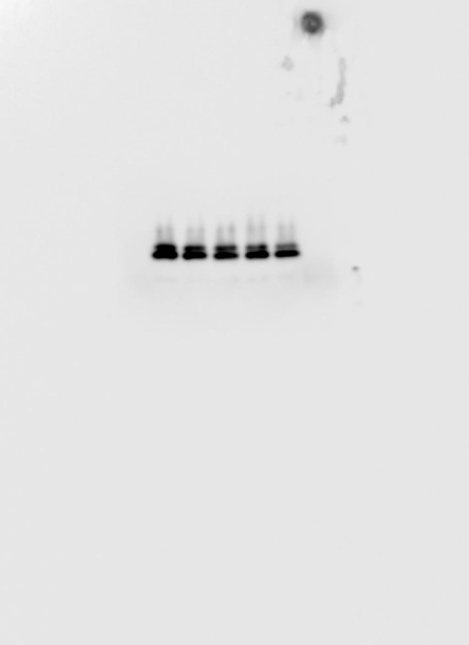
**

**Fig.5I p-AKT**

**
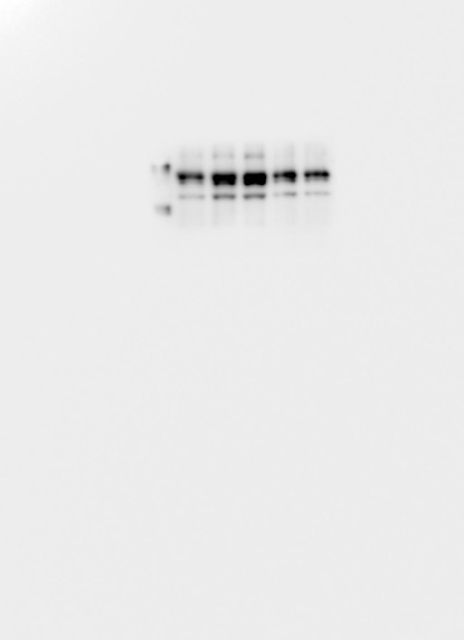
**

**Fig.5I p-ERK**

**
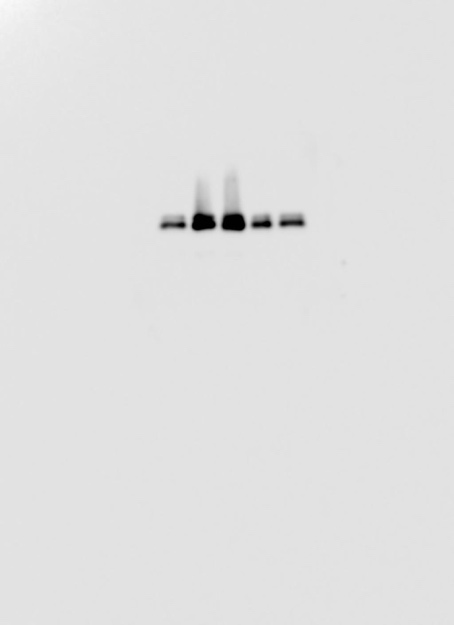
**

**Fig.5I SOS1**

**
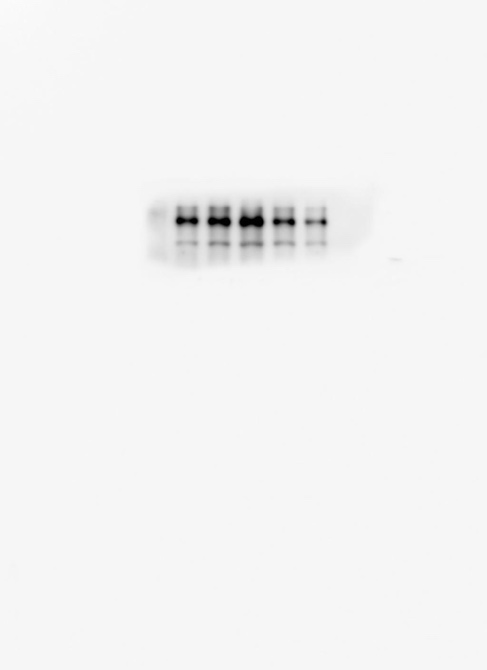
**

**Fig.5I β-Actin**

**
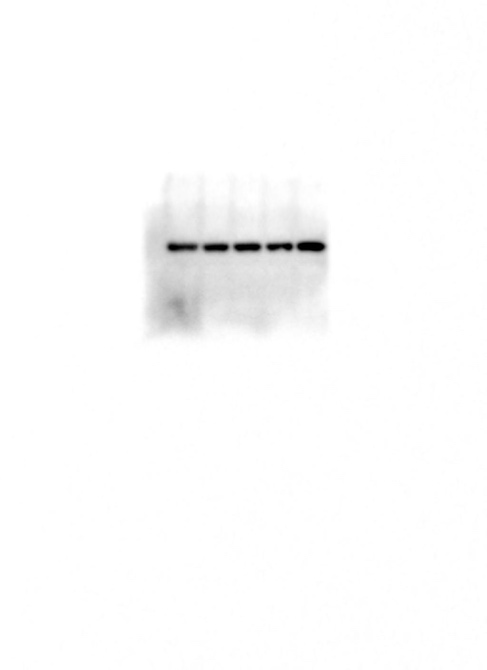
**

**Fig.7C β-Actin**

**
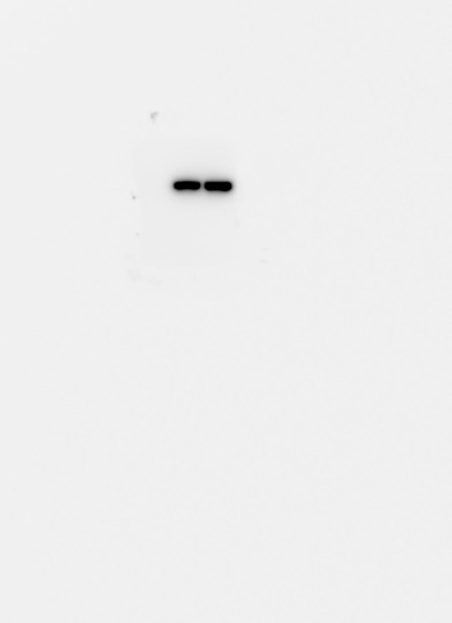
**

**Fig.7C EIF4A3**

**
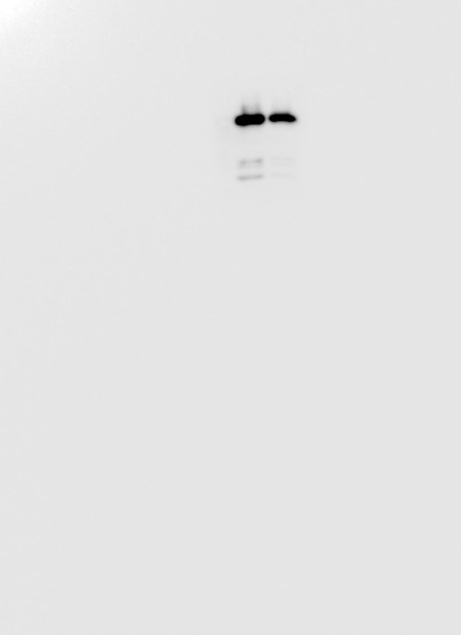
**

**Fig.8E AKT**

**
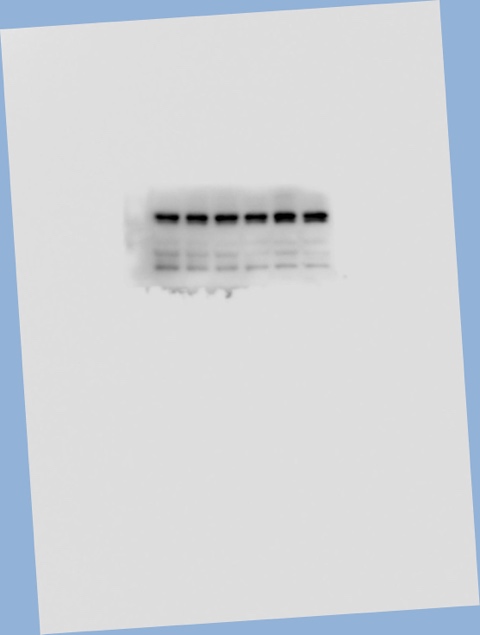
**

**Fig.8E ERK**

**
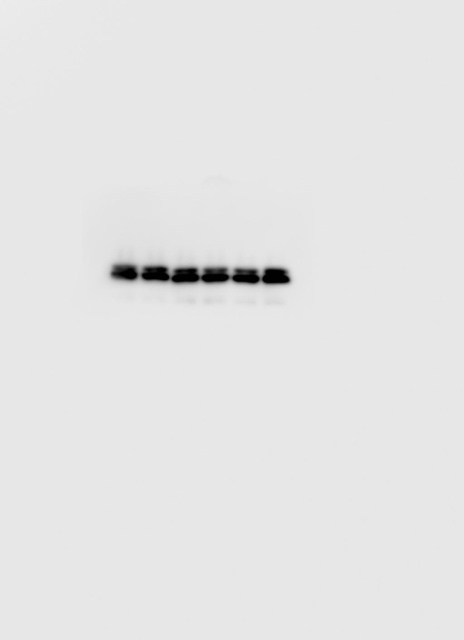
**

**Fig.8E p-AKT**

**
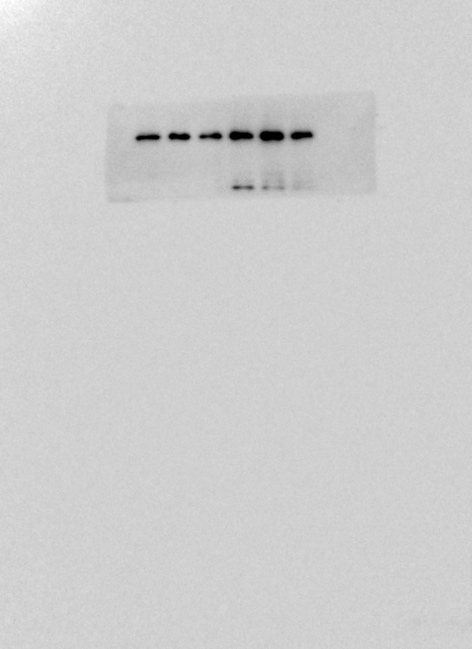
**

**Fig.8E p-ERK**

**
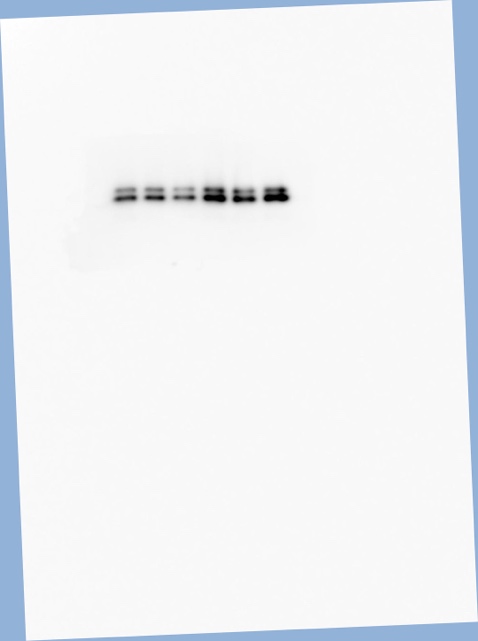
**

**Fig.8E SOS1**

**
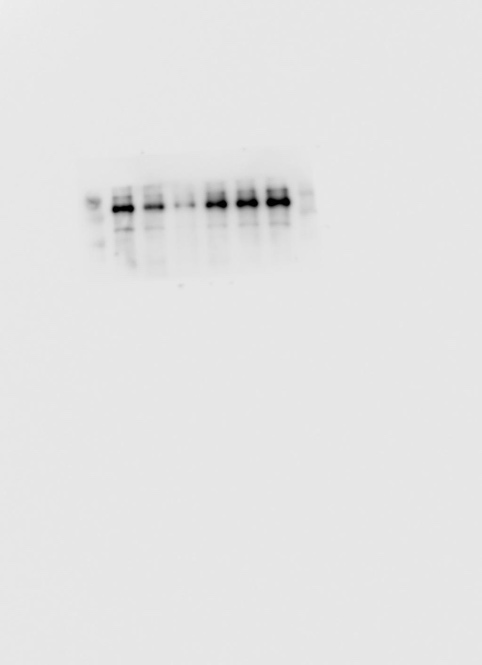
**

**Fig.8E β-Actin**

**
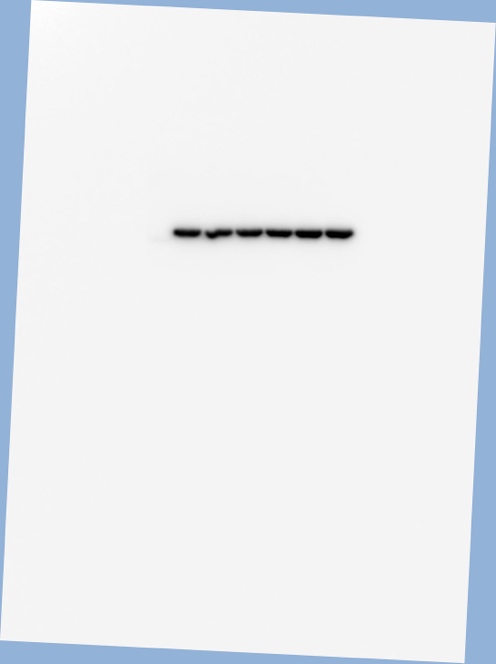
**
